# Targeting *Candida albicans O*-acetyl-L-homoserine sulfhydrylase (Met15p) in antifungal treatment

**DOI:** 10.1038/s41598-024-79886-y

**Published:** 2024-11-15

**Authors:** Aleksandra Kuplińska, Kamila Rząd, Joanna Stefaniak-Skorupa, Katarzyna Kozłowska-Tylingo, Marek Wojciechowski, Sławomir Milewski, Iwona Gabriel

**Affiliations:** 1 Department of Pharmaceutical Technology and Biochemistry, Faculty of Chemistry, Gdansk University of Technology, 11/12 Narutowicza Str, Gdansk, 80-233 Poland; 2 Department of Organic Chemistry, Gdansk University of Technology, Gdansk, Poland

**Keywords:** Enzymes, Antimicrobials, Fungi, Medicinal chemistry, Target identification, Target validation

## Abstract

Fungal infections are a serious threat to public health as they are becoming increasingly frequent. A major problem stems also from a rising fungal resistance to currently available antifungal therapies, therefore novel molecular targets are highly desirable. Exploration of enzymes participating in the biosynthesis pathways of essential amino acids such as L-methionine (L-Met) may provide new insights into pharmaceutical development. The *MET15* gene from *Candida albicans*, encoding *O*-acetyl-L-homoserine sulfhydrylase (Met15p), an enzyme catalyzing the second step in that pathway, was cloned and expressed in two versions: as N and C-terminal oligo-His-tagged fusion proteins. The recombinant enzymes revealed appropriate activity, and catalyzed conversion of *O*-acetyl-L-homoserine and a sulfide ion to produce L-homocysteine. A new RP-HPLC-DAD method, using the enzymatic reaction product pre-column derivatization with 5,5’-dithio-bis-(2-nitrobenzoic acid) was developed and used by us to determine Met15p activity. Newly synthesized compounds as well as two commercially available exhibited a Met15p inhibitory effect which was related to antifungal activity. Fungal cells’ sensitivity to inhibitors depending on the presence or absence of L-Met in the medium clearly indicated Met15p targeting. Moreover, the synergistic effect of the first methionine biosynthetic enzyme affecting inhibitor and Met15p inhibitors indicate that methionine biosynthesis pathway enzymes are promising molecular targets.

**Supplementary Information:**

The online version contains supplementary material available at 10.1038/s41598-024-79886-y.

## Introduction

In 2022 The World Health Organization launched the first priority fungal pathogens list, which categorizes *Candida albicans* together with *Candida auris*,* Cryptococcus neoformans*, and *Aspergillus fumigatus*into the critical group of human pathogenic fungi^1^. Invasive fungal diseases (IFDs) caused by these pathogens are associated with the highest incidence and mortality rates. The development of modern medicine and facilitated availability of treatment has led to an increase in the number of cases of IFDs, as the at-risk population is expanding. Patients affected by IFDs include most often transplant recipients or cancer patients but also those suffering from diabetes, asthma, or influenza^2^. Recently, IFDs have been found associated with COVID-19 patients and survivors^2–4^. A major threat is also caused by the rising resistance of fungal micro-organisms to the scarce available drugs. Existing four classes of antifungal drugs used in the clinical treatment of IFDs, namely echinocandins, azole derivatives, 5-fluorocytosine and polyene macrolide antibiotics, target either one of the enzymes participating in β(1→3)-glucan, ergosterol or DNA biosynthesis or ergosterol present in the fungal cell membrane, respectively. Chemotherapies with available antifungal drugs are often associated with adverse effects, drug-drug interactions, and need for a prolonged treatment^5^. Novel antifungal drug candidates such as rezafungin or ibrexafungerp offering enhanced efficacy and safety in in-vivo and murine models, are not regarded as an alternative therapeutic option for the treatment of IFDs as they display a comparable spectrum to echinocandins^5^.

Regardless of the rising concern, the role of IFDs as a challenging global health problem receives inadequate appreciation and progress in discovery and development of novel antifungal drugs seems insufficient^6^. The development of new antifungal drugs is focused on the improvement of properties of existing antifungal medicines, however, some of the drug candidates exhibit novel mechanisms of action due to the interaction with still unexploited targets, such as of heat shock protein 90 or calcineurin signaling^6^. One of the possibilities in this respect are the enzymes catalyzing particular steps in biosynthetic pathways of amino acids, present in fungal cells, not in humans^7^. Fungi-specific pathways of L-methionine (L-Met) or L-tryptophan (L-Trp) biosynthesis seem to be particularly interesting^7^. These two amino acids are present in human serum at relatively low concentrations^8^, not sufficient to rescue methionine or tryptophan auxotrophy of fungal cells resulting from inhibition of L-Met or L-Trp biosynthesis. Recently, we have shown that L- and D-penicillamine are inhibitors of C. *albicans* L-homoserine *O*-acetyltransferase (CaMet2p), an enzyme that catalyzes the first committed step of methionine biosynthesis. Both enantiomers of penicillamine exhibited antifungal in vitro activity and their growth inhibitory effect could be abolished only upon addition to the growth medium of L-Met at concentration at least 10-fold higher than the physiological level of this amino acid in human serum^9^. That finding clearly confirmed a potential utility of Met2p as an antifungal target, previously suggested by Nazi et al. and Seyran^10,11^.

**Fig. 1:**
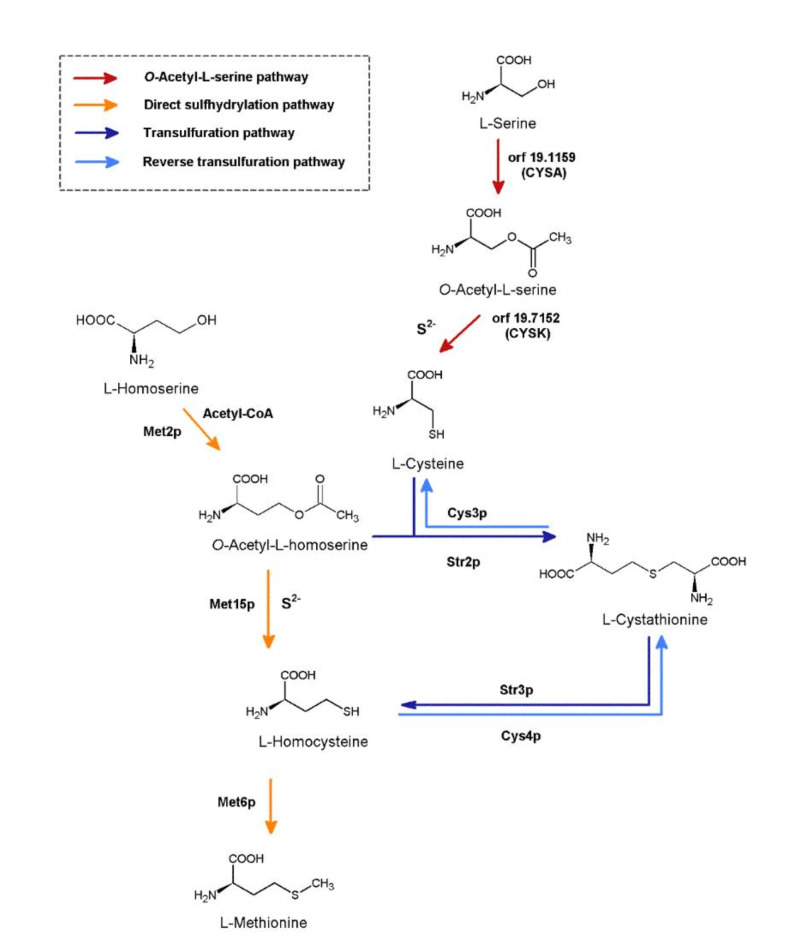
Putative pathways of L-methionine biosynthesis in *C. albicans* cells. Homoserine *O*-acetyltransferase EC 2.3.1.31 (Met2p); bifunctional *O*-acetyl-L-homoserine/*O*-acetyl-L-serine sulfhydrylase EC 2.5.1.49, EC 2.5.1.47 (Met15p); methionine synthase EC 2.1.1.13 (Met6p); cystathionine-β-synthase EC 4.2.1.22 (Cys4p); cystathionine-β-lyase EC 4.4.1.8 (Str3p); cystathionine-γ-lyase EC 4.4.1.1 (Cys3p); cystathionine-γ-synthase EC 2.5.1.48 (Str2p); putative homologue of the Aspergillus nidulans L-serine *O*-transacetylase, orf19.1159 (CYSA); putative homolog of *Aspergillus nidulans O*-acetyl-L-serine sulfhydrylase, orf19.7152 (CYSK).

The biosynthesis of L-Met in fungal cells can proceed through two ways utilizing different substrates, namely from L-homoserine *via* the direct sulfhydrylation pathway and from L-cysteine derived from L-serine *via* the transsulfuration pathway (Fig. 1). Filamentous fungi as well as most yeasts, including *C. albicans*, possess both pathways in full versions. However, *S. cerevisiae* and *C. glabrata* lack the *O*-acetyl-L-serine pathway, so that in this yeast the transsulfuration pathway can be fed exclusively by exogenous L-cysteine^12^.

**Fig. 2:**
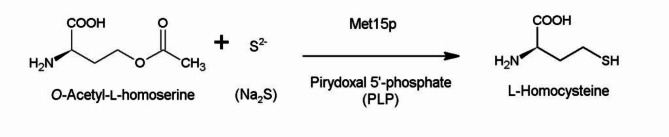
Reaction catalyzed by Met15p.

One of the enzymes that participate in L-Met biosynthesis is the *O*-acetyl-L-homoserine sulfhydrylase (Met15p), which catalyzes conversion of *O*-acetyl-L-homoserine (OAH) into L-homocysteine (HCT) with a sulfide ion as a sulfur donor (Fig. 2).

Met15p is involved in the direct sulfhydrylation pathway (Fig. 1), however, there is some evidence that at least in some fungi this enzyme can also utilize *O*-acetyl-L-serine (OAS) as a substrate, to produce L-cysteine, used in the transsulfuration pathway. A Met15p version able to utilize both OAH and OAS is called the bifunctional *O*-acetyl-L-homoserine/*O*-acetyl-L-serine sulfhydrylase (EC 2.5.1.49, EC 2.5.1.47). Met15p is a pyridoxal 5-phosphate (PLP) dependent enzyme belonging to the γ-elimination subclass of the Cys/Met metabolism PLP-dependent family of enzymes^13^. This enzyme has not been yet considered as a potential target for antifungals. As mentioned above fungal cells differ in their presence of both pathways. *C. guilliermondii* and *C. albicans*mutant cells deficient in Met15p activity exposed prototrophic character in the absence of L-methionine or sulfate in the medium, respectively^14,15^. This indicated the presence of a transsulfuration pathway that can rescue the Met15p deficiency. On the other hand, *C. glabrata* and *Saccharomyces cerevisiae*mutant cells, depleted in the Met15p encoding gene, revealed auxotrophy for L-Met or sulfur^16,17^. The explanation for the greater sensitivity of *C. glabrata* and *S*. *cerevisiae* is the absence of the pathway leading to L-cysteine production from *O*-acetyl-L-serine, the *O*-acetyl-L-serine pathway^12,18^. Our previously published results also indicated that L-penicillamine, a relatively weak inhibitor of Met2p (Fig. 1), exhibited antifungal activity only against *C. glabrata* and *S. cerevisiae*^9^. That proves that *C. glabrata* as well as *S. cerevisiae* lack functional L-cysteine biosynthesis pathway from *O*-acetyl-L-serine (Fig. 1) meaning that it can be only synthesized by the reverse transsulfuration pathway from L-methionine.

In this paper, we present the results of our studies on identification and cloning of the *C. albicans MET15* gene, as well as construction, isolation, and characterization of CaMet15p oligoHis-tagged forms. Several *O*-acetyl-L-homoserine or L-homocysteine structural analogs were analyzed as potential inhibitors of CaMet15p. In these studies, a newly developed RP-HPLC-MS method employing pre-column derivatization of L-homocysteine with 5,5’-dithio-bis-(2-nitrobenzoic acid) (DTNB) was used. The identified inhibitors of CaMet15p were also tested for their antifungal in vitro activity, as single agents and in combination with inhibitors of CaMet2p, looking for the possibility of synergistic growth inhibitory effect.

## Results and discussion

### Gene identification

The *MET15* gene (orf19.13090), encoding putative *C. albicans O*-acetyl-L-homoserine sulfhydrylase (CaMet15p) was retrieved from the *Candida*Genome Database^19^. The deduced amino acid sequence of CaMet15p exhibited 71% identity to that of the bifunctional *O*-acetyl-L-homoserine/*O*-acetyl-L-serine sulfhydrylase from *S. cerevisiae* but 50% and 46% identity with the sequence of bacterial *O*-acetyl-L-homoserine sulhydrylases from *Wolinella succinogenes* and *Mycobacterium marinum*, respectively. A multiple sequence alignment shown in Figure S1revealed that CaMet15p contains all counterparts of the active site residues participating with their side chains functionalities in PLP binding, previously identified in available crystal structures of its yeast and bacterial homologs, including the catalytic Lys207, presumed to form a Schiff base linkage with PLP prior to substrate binding. CaMet15p is a 439 aa long polypeptide with pI value of 5.59 and a theoretical MW of 48.8 kDa (Prot-Param analysis^20^).

#### CaMet15p – subcloning, protein expression and purification

**Fig. 3:**
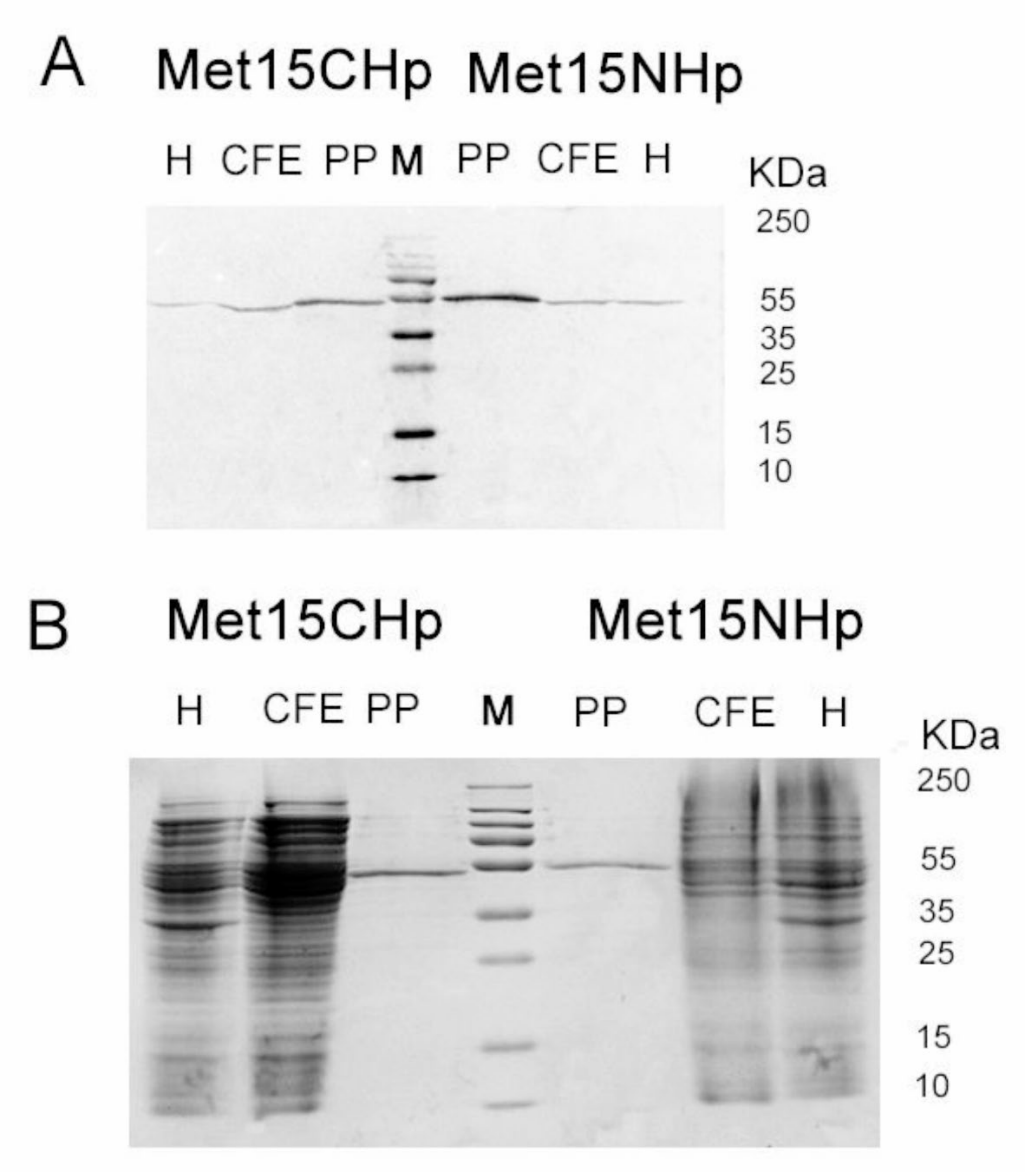
(**A**) Western Blot analysis of CaMet15NHp and CaMet15CHp purification; (**B**) SDS-PAGE electrophoresis analysis of Met15NHp and Met15CHp purification. Electrophoresis 18 V cm^−1^ 10% gel; H, harvest from protein overproduction; CFE, cell-free extract; PP, purified protein; M, Thermo Scientific PageRuler™ Plus Prestained Protein Ladder. The gels were cut to increase the clarity of the presentation. The unedited gels are shown in Supplementary Materials.

Vector pLate11 for ligation independent cloning (LIC) and tightly regulated bacterial expression of an untagged protein was used to subclone *CaMET15* gene and obtain expression system for wild-type CaMet15p. The level of heterologous expression of the wild-type *CaMET15* gene and CaMet15p overproduction was very low (data not shown), causing purification of this protein highly troublesome. We have thus constructed expression vectors ensuring overproduction of CaMet15p as N- and C-oligoHis-tagged versions. For this purpose, the *CaMET15* gene was cloned into pLate52 and pLate31 vectors. Both Met15NHp and Met15CHp were expressed in *Escherichia coli* ER2566 host cells, with production level of 3% and 5% of the total protein pool, respectively. Production of the target proteins was confirmed by SDS-PAGE (Fig. 3B) and Western Blot analysis (Fig. 3A), resulting in the formation of a band corresponding to ~ 58 kDa. CaMet15NHp and CaMet15CHp were purified using HisTrap^TM^ Fast Flow column in AKTA Fast Protein Liquid Chromatography system, with a final purity of 90% and 93%, respectively (Figure S2).

### Development of a novel method of Met15p activity determination

**Fig. 4:**
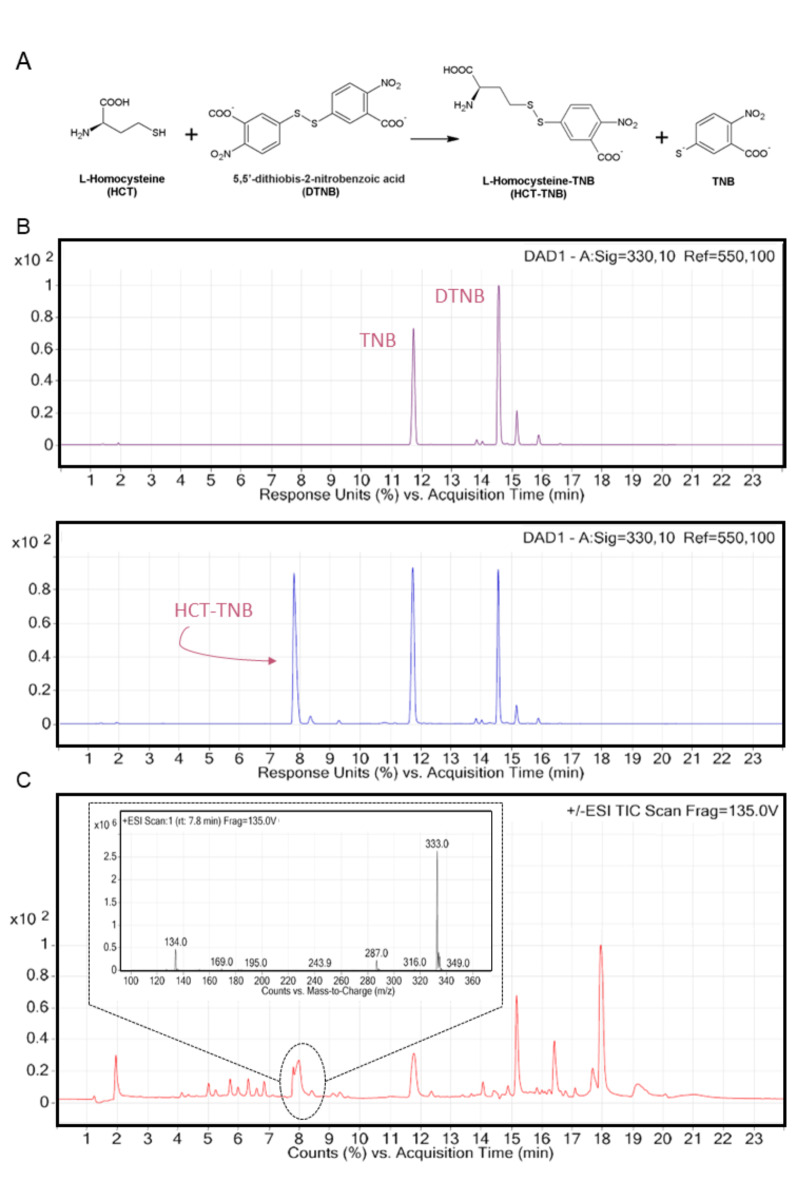
(**A**) Pre-column derivatization of L-homocysteine with DTNB. (**B**) Chromatograms of components of the control (upper) as well as enzymatic (lower) reaction mixtures. The reaction mixture contained 25 mM Tris-HCl pH 8.0; 100 mM NaCl; 10 mM *O*-acetyl-L-homoserine; 0.1 mM Na_2_S; and 0.2 mM PLP. The reaction was started by the addition of 10 nM pure enzyme. The control mixture contained all ingredients except the substrate *O*-acetyl-L-homoserine. (**C**) Mass spectrometry (MS) analysis of the enzymatic reaction (r.t 7.8 min). m/z: [HCT-TNB + H]^+^ 333.0.

The previously used methods of Met15p activity determination based on selective quantification of L-homocysteine formed in the reaction catalyzed by the enzyme. One of them employed the use of radioactive sulfide(^35^S)^21^ and in another one, a highly toxic HgCl_2_is used^22^. Aiming to avoid the use of radioactive or toxic reagents we decided to develop a new method for the assessment of Met15p activity, via the RP-HPLC-DAD detection of L-homocysteine, pre-column derivatized with DTNB to form HCT-TNB (Fig. 4A).

As indicated in Fig. 4B, C we were able to identify the HCT-TNB reaction product by RP-HPLC-DAD method and verify its molecular mass by mass spectrometry analysis. The change in the surface area under the peak corresponding to HCT-TNB that we observed, depending on the concentration of the OAH substrate, allowed us to effectively determine the enzyme activity (Table S1). Examples of chromatograms used to measure the reaction rate of CaMet15p are shown in Figure S3.

### Basal properties of the recombinant enzyme

**Fig. 5:**
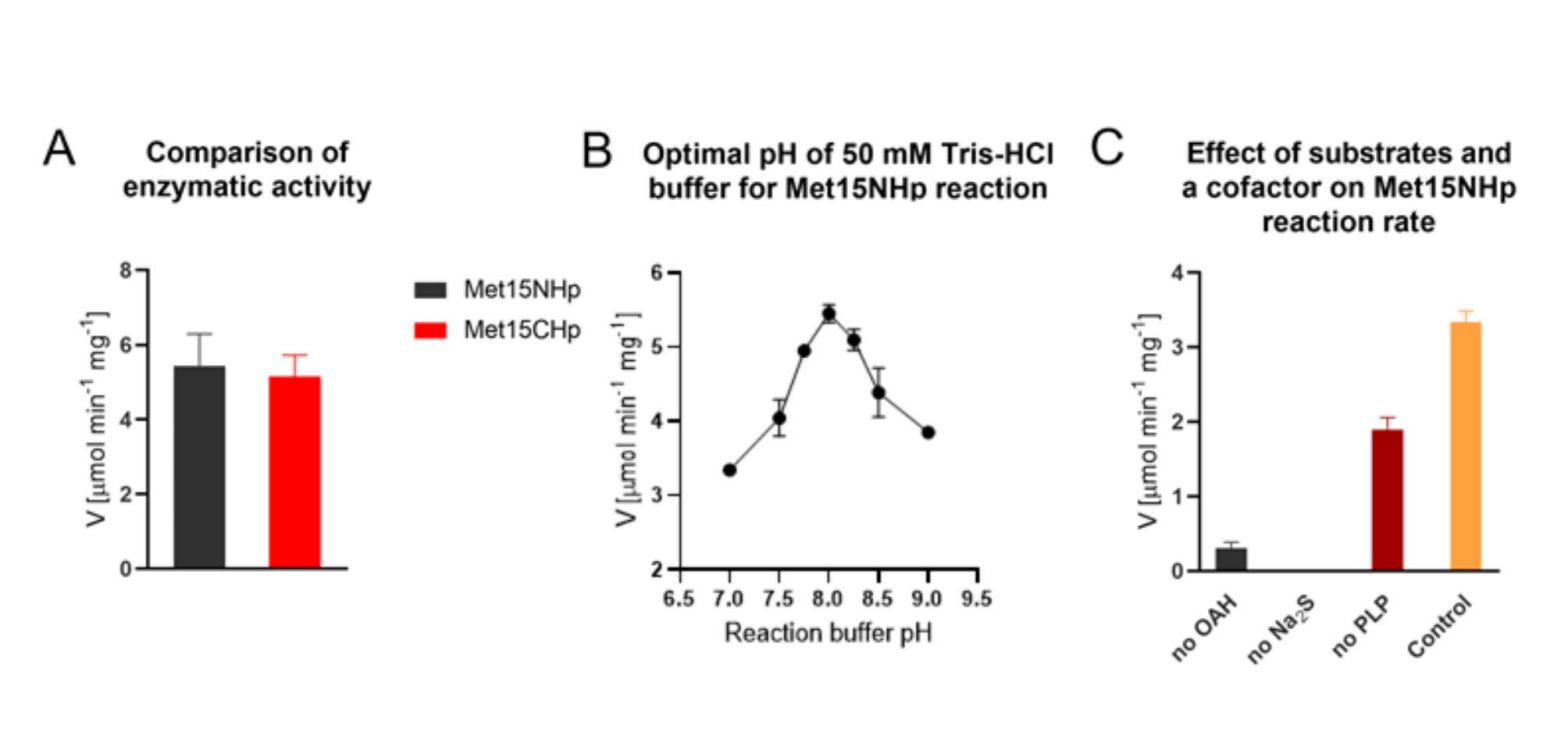
(**A**) Comparison of rate of the reaction catalyzed by CaMet15NHp and CaMet15CHp; (**B**) Determination of CaMet15NHp optimal pH for maximal activity; (C) Influence of absence of substrate (OAH or Na_2_S) or a cofactor on the reaction rate, compared to the control reaction which includes all substrates and a cofactor.

The purified CaMet15NHp, as well as CaMet15CHp, displayed an activity of *O*-acetyl-L-homoserine sulfhydrylase, catalyzing formation of L-homocysteine (HCT) from *O*-acetyl-L-homoserine (OAH), with Na_2_S as a source of sulfide ion and a PLP as a cofactor (Fig. 2). This activity was comparable for both oligoHis-tagged enzyme versions (Fig. 5A), thus suggesting that presence of an oligoHis tag at either N- or C-end does not affect this activity. The pH optimum of the enzyme activity was observed at 8.0 (Fig. 5B). To verify the potential ability of CaMet15p to utilize *O*-acetyl-L-serine (OAS) as a substrate we have performed additional experiments. In our hands, CaMet15NHp did not exhibit the bifunctional *O*-acetyl-L-homoserine/*O*-acetyl-L-serine sulfhydrylase activity, as no L-Cys production was detected when *O*-acetyl-L-serine was present in the reaction mixture instead of OAH. Our results are in contrast with the enzyme counterpart from *S. cerevisiae*, which was found bifunctional^23^. On the other hand, mono-functional *O*-acetyl-L-homoserine sulfhydrylase was demonstrated in *Schizosaccharomyces pombe*, containing two types of Met15p enzymes, one of which reacting only with OAH but not OAS^21,24^. Other Met15p enzymes found in *B. flavum* and *T. thermophilus*displayed the bifunctional activities that were significantly shifted towards using OAH as preferred substrate, with a relative enzymatic activity of up to 5% observed when utilizing OAS^25,26^.

Further studies on enzyme properties were performed with only one version of the recombinant enzyme, namely CaMet15NHp. There is little doubt that a substantial part of the purified CaMet15NHp contained bound PLP, since its activity in the reaction mixture lacking PLP was ∼50% lower than that in the presence of 0.2 mM PLP (Fig. 5C) Determination of kinetic parameters of CaMet15NHp-catalyzed reaction revealed a substantial difference in enzyme affinity to OAH and Na_2_S. Determined K_m_ and V_max_ values for OAH were 9.12 ± 1.77 mM and 15.50 ± 0.81 µmol min^−1^ mg^−1^, respectively, while those for Na_2_S equaled to 0.027 ± 0.0021 mM and 8.07 ± 0.12 µmol min^−1^ mg^−1^, respectively (Figure S4). This is not very surprising since similar values were previously found for *S. pombe* Met15p, with K_m_equal to 12.5 mM and 0.0530 mM, for OAH and sulfide, respectively^27^. However, for the *Thermus thermophilus HB8* enzyme, the K_m_values were 6.80 mM for OAH and 1.30 mM for sulfide^26^ and for the *Clostridioides difficile*Met15p determined parameters were 0.6 mM and 0.4 mM, respectively^28^. This may suggest that a high affinity for the sulfide donor substrate, reflected by the low K_m,_ is a property of the yeast but not bacterial counterpart of the enzyme.

CaMet15NHp was stable for 22 h after purification when stored at 4 °C, however, supplementation with 20% (v/v) of glycerol helped maintaining enzyme stability, so that it could be stored at 4 °C for at least two days without a significant loss of activity (Figure S5).

### Molecular mass and oligomeric structure of CaMet15NHp

The molecular mass of a single subunit of the *C. albicans MET15*gene product, calculated with the help of the ProtParam^20^ was 48.8 kDa, which is comparable with the molecular mass of purified CaMet15NHp determined by the SDS-PAGE electrophoresis, resulting in a single band of 48.2 ± 1.7 kDa (Figure S6). The molecular mass of a native form of the enzyme was determined by size exclusion chromatography (SEC) and native-PAGE. The SEC determination revealed MW = 188.4 ± 4.6 kDa, which suggests a homotetrameric structure of the enzyme. This was confirmed by the results of the native-PAGE, showing a prominent band of approximately 203.3 ± 1.3 kDa and a faint band of 106.7 ± 0.94 kDa (Figure S6). It can be thus concluded that CaMet15NHp exists mostly as a homotetramer but its homodimer form is also possible. The quaternary structure of CaMet15p is therefore consistent with those of its *S. cerevisiae*^29^ (PDB code: 8OVH), *Wolinella succinogenes*^13^ (PDB code: 3RI6), and *Mycobacterium marinum*^30^ (PDB code: 4KAM) counterparts.

### Screening for inhibitors of CaMet15p

**Fig. 6:**
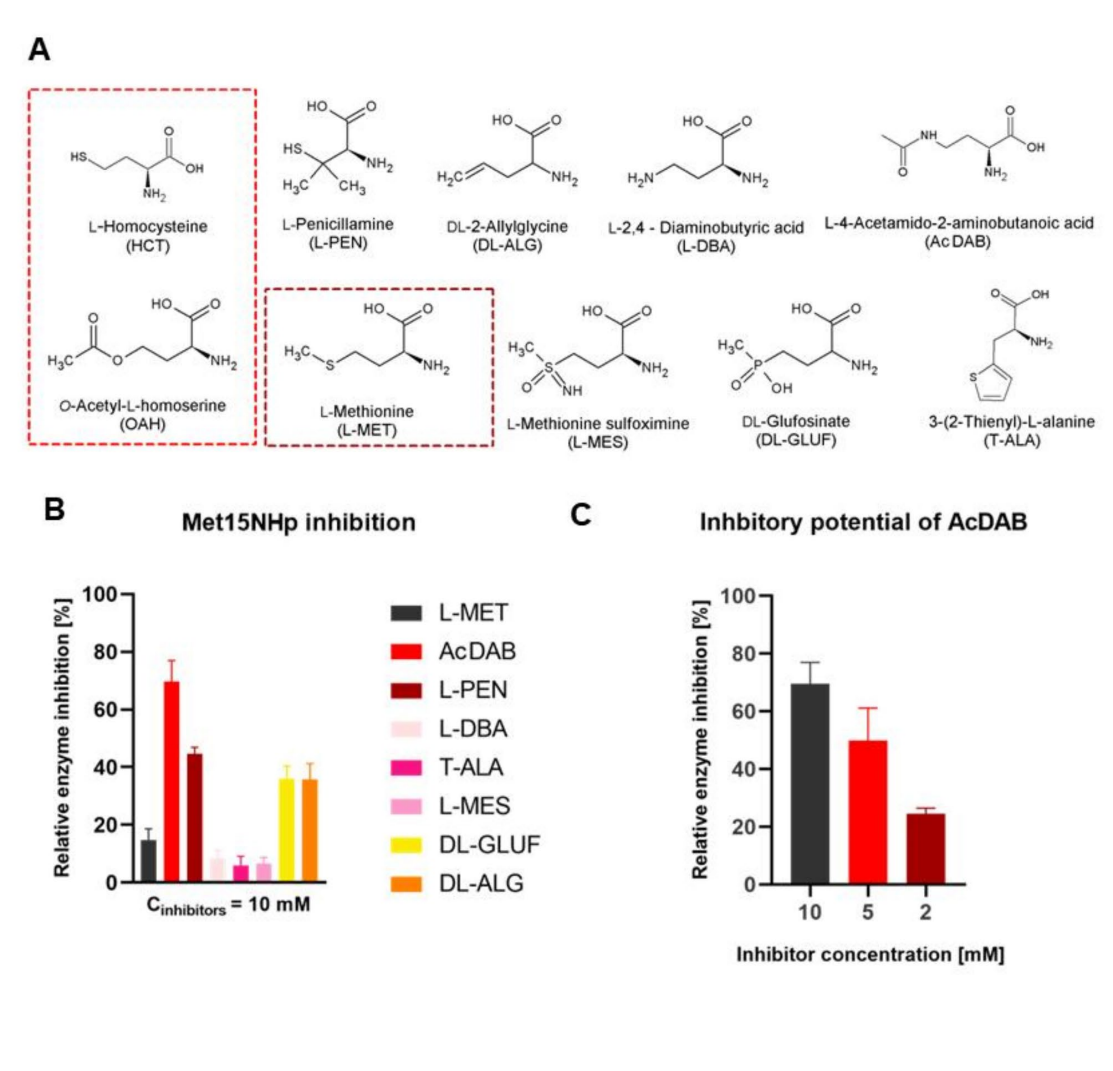
Inhibition of CaMet15NHp activity. (**A**) Structures of potential inhibitors. (**B**) Inhibition of enzyme activity by OAH, HCT and L-Met analogs at 10 mM concentration. (**C**) Concentration-dependent inhibitory potential of AcDAB.

The inhibitory activity towards CaMet15NHp of seven potential inhibitors at 10 mM concentration was tested. All these compounds can be considered structural analogs of OAH, HCT, or L-Met (Fig. 6A). Data presented in Fig. 6B indicate that two out of seven compounds tested, namely L-penicillamine (L-PEN) and L-4-acetamido-2-aminobutanoic acid (AcDAB) (Figure S7-S9), inhibited enzyme activity in at least 50%. Another two, DL-2-allyglycine (DL-ALG), and DL-glufosinate (DL-GLUF) were slightly inhibitory and the remaining three were inactive. In the case of ALG and GLUF, used as racemic mixtures, one may expect that their pure L enantiomers could be more efficient as enzyme inhibitors. This is worth mentioning that L-PEN was previously reported by us as an effective inhibitor of *C. albicans* L-homoserine *O*-acetyltransferase (Met2p), i.e. an enzyme that catalyzes the first committed step of methionine biosynthesis. AcDAB appeared as the most effective inhibitor of CaMet15NHp, which inhibited ∼70% of enzyme activity at 10 mM, 53% at 5 mM and 25% at 2 mM. (Fig. 6C). The inhibitory potential of this compound against Met15p was previously demonstrated for the bacterial version of the enzyme^28^.

Additionally, we tested whether CaMet15NHp was inhibited by L-Met, i.e. the end-product of the L-methionine biosynthetic pathway. The inhibition level of L-Met at 10 mM was marginal (less than 15%, Fig. 6B), thus suggesting that regulation of CaMet15p activity through the feedback inhibition is unlikely.

### Molecular modeling of AcDAB binding and a rational design of a potentially more effective inhibitor

**Fig. 7:**
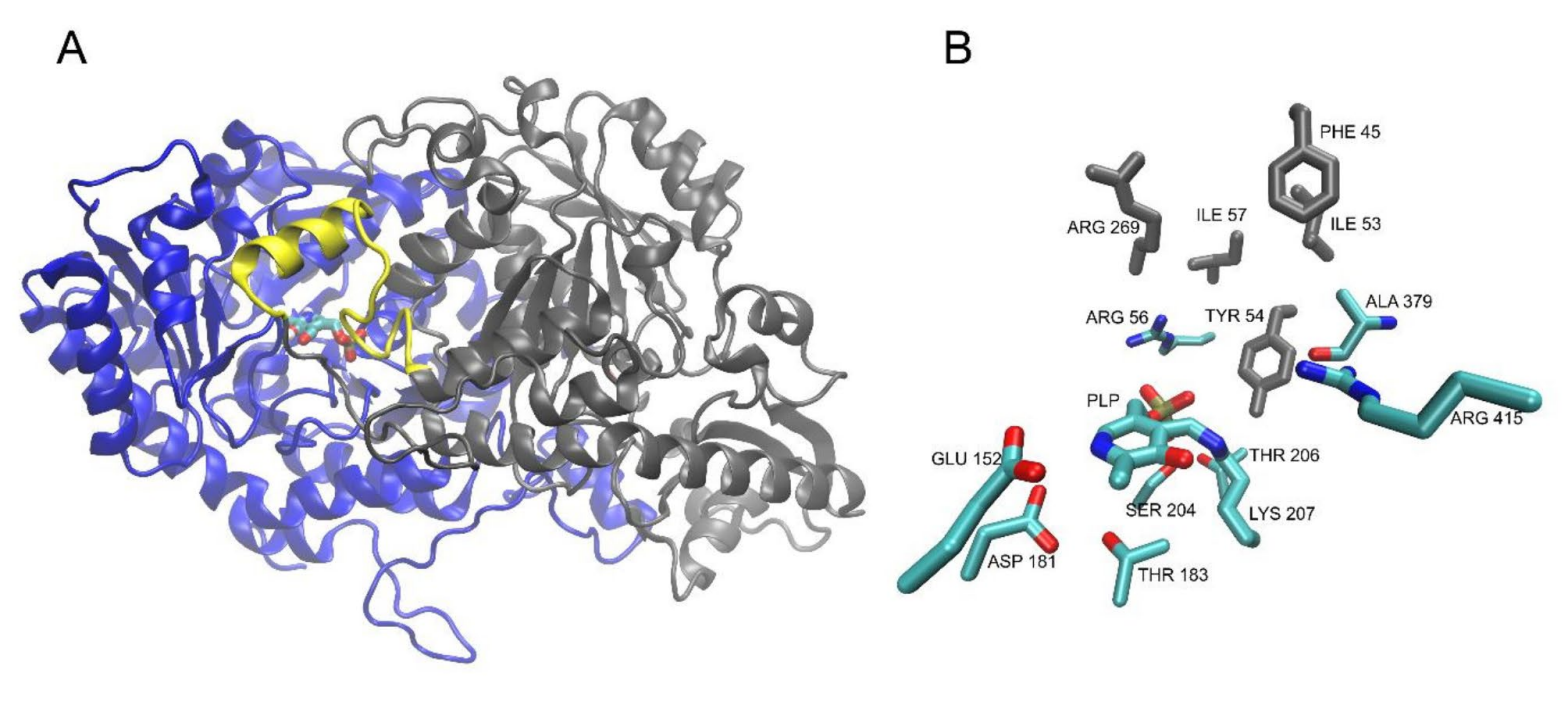
(**A**) Structure of the modeled CaMet15p. The flexible loop of residues 36–60 covering the binding site is depicted in yellow, while the PLP molecule present in the binding site is shown as sticks. (**B**) The active center amino acid residues of CaMet15p. The PLP molecule and conserved residues marked in Figure S1 are depicted as thin sticks. Residues forming the hydrophobic pocket are shown as thick gray sticks.

The AcDAB binding at the enzyme active site was investigated by molecular modeling and ligand docking studies. Since an X-ray structure of *C. albicans O*-acetyl-L-homoserine sulfhydrylase is not known, the structure of Met15p from *S. cerevisiae*(PDBID: 8OVH) was used as a template to prepare the AlphaFold^31^ model of the *C. albicans* enzyme. The structure of the modeled homodimer receptor is presented in Fig. 7A. Conserved amino acids indicated in Figure S1, involved probably in PLP binding or forming the nearby substrate binding site and a flexible loop covering it, are presented in Fig. 7B.

**Fig. 8:**
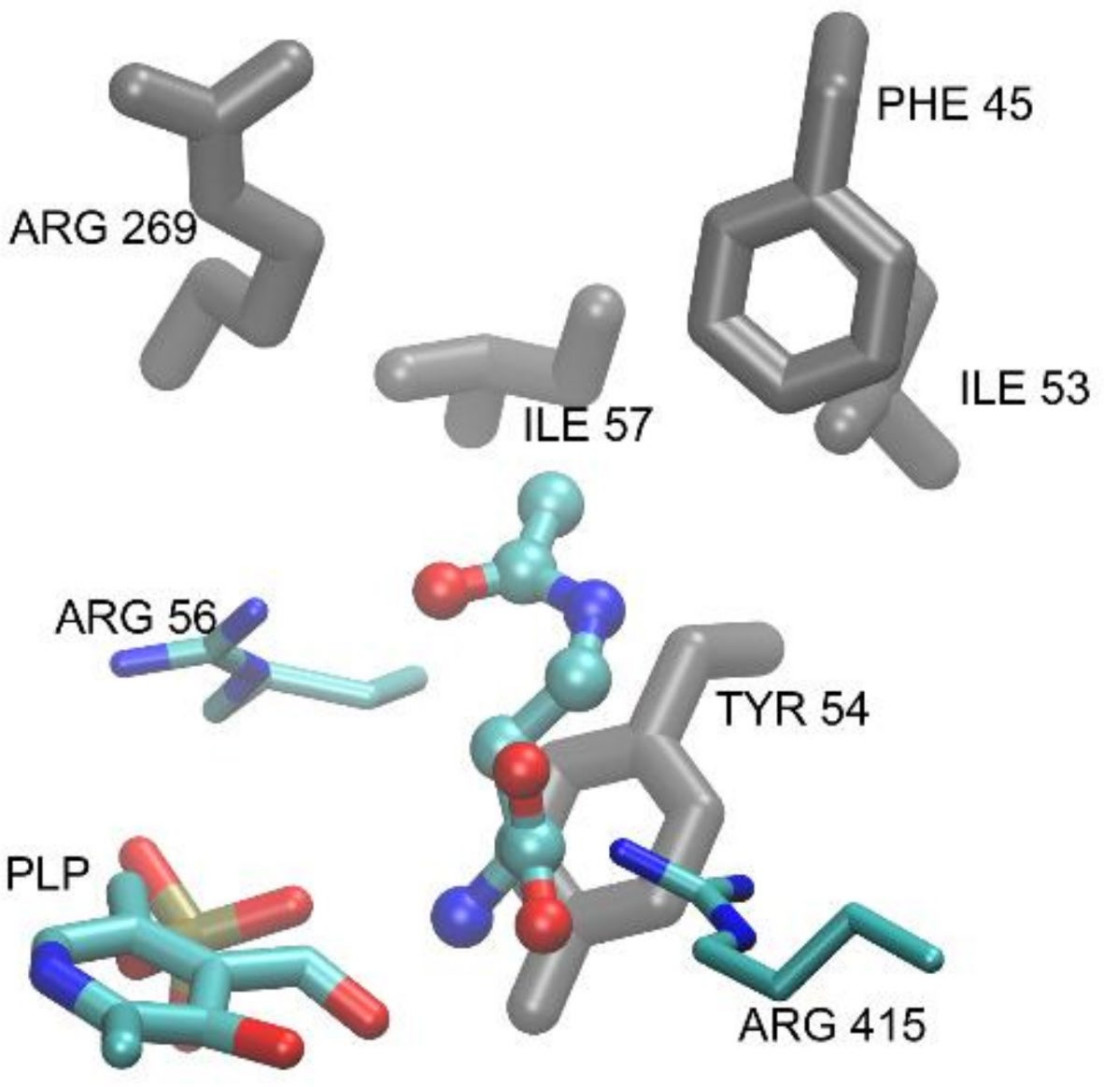
Result of a regular docking of AcDAB. The inhibitor is shown as a balls and sticks model, while the PLP molecule and essential residues are depicted as thin sticks. Residues forming the hydrophobic pocket are shown as thick gray sticks.

The mechanism of catalysis performed by Met15p is complex and involves the formation of covalent bonds between a substrate, i.e. OAH and the PLP cofactor^32^. Although the bond formation phenomena are out of the scope of the regular docking simulations, its first step, namely the formation of a noncovalent ligand-receptor complex, as well as the conformation of the covalent intermediate, could be modeled and thus, the essential residues and interactions involved in the process still could be identified. To achieve this, docking of the substrate (OAH) and the most effective inhibitor (AcDAB) was performed. The result obtained for AcDAB is shown in Fig. 8.

Unfortunately, there is no experimental structure of this or any similar complex available for comparison. However, since during the catalytic reaction, the α-amino group of AcDAB is expected to form a Schiff base with PLP^32^ involving its aldehyde moiety, and in the resulting docked poses the two groups were found to be in close proximity, this result is considered reasonable. It is additionally supported by the fact that the carboxyl moiety of the ligand points toward nearby conserved Arg415 residue, further contributing to the stabilization of the complex. An identical pattern of interactions involving ligands’ carboxyl and α-amino groups was also identified in the results of docking of other tested inhibitors, namely L-PEN, L-GLUF and L-ALG), additionally confirming the validity of the model (detailed data not shown).

**Fig. 9:**
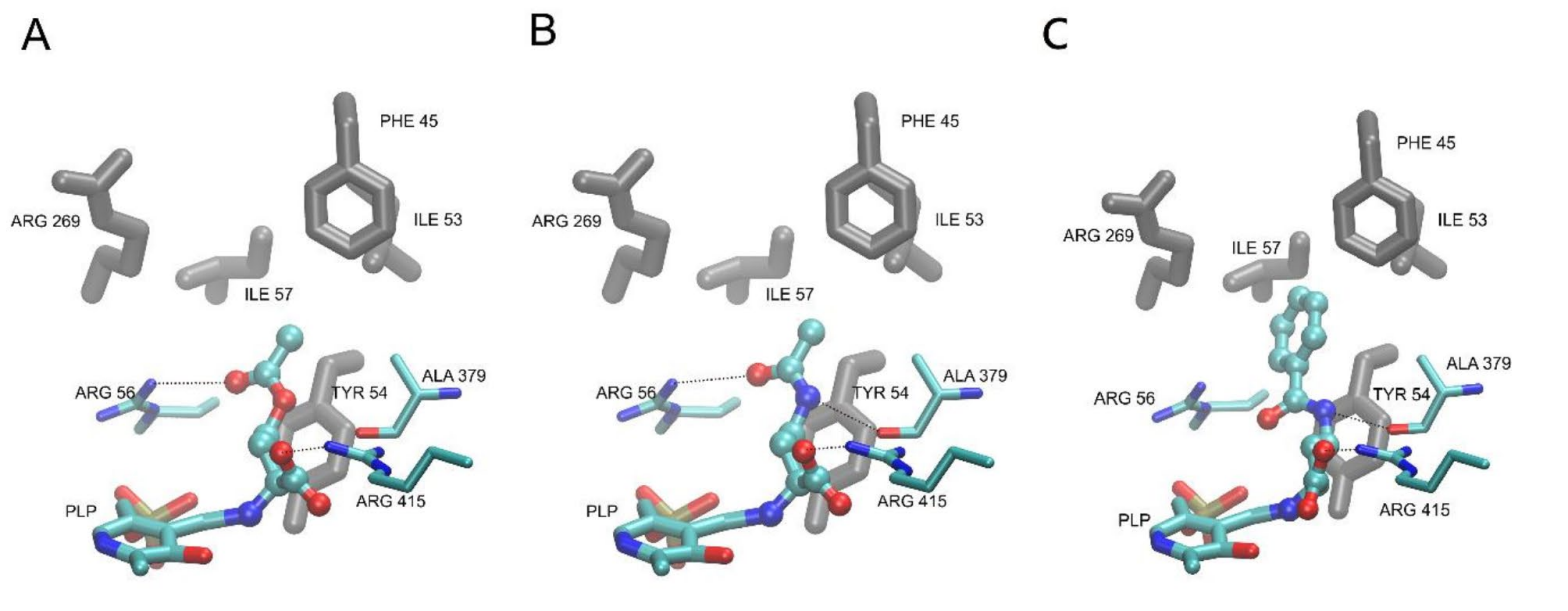
Results of molecular docking of the substrate OAH (**A**), AcDAB (**B**) and (**C**) BzDAB at the active site of CaMet15p. The ligands are shown as balls and sticks models, while the PLP molecule and the active site essential residues mentioned in the text are depicted as thin sticks. Residues forming the hydrophobic pocket are shown as thick gray sticks. Hydrogen bonds are shown as dashed lines.

It should be noted that structures of OAH, i.e. enzyme substrate and its most potent inhibitor, AcDAB, are very similar, with the only difference being the substitution of the ester group in OAH with the amide group in AcDAB. Due to AcDAB inhibitory effectiveness this replacement resulted in a much higher affinity of the inhibitor molecule for the enzyme. To shed some light on the possible source of this affinity difference at the molecular level, models of docked complexes for both ligands were studied. As mentioned earlier, the entrance to the binding site of Met15p is covered by the flexible loop (Fig. 7A) composed of residues 36–60, some of which (Phe45, Ile53, Tyr54, Ile57), along with the nonpolar part of the Arg269 side chain, form a small hydrophobic pocket. It turned out that in the resulting complexes of both ligands, the methyl group of the acetamide/acetate moiety points toward this pocket while its carbonyl group forms an additional hydrogen bond with Arg56, stabilizing the bound conformations. On the other hand, in the bound pose, the flat amide of AcDAB is parallel to the Tyr54 phenyl ring, with the amide NH proton pointing toward the nearby backbone carbonyl of Ala379 (Fig. 7B). This is worth mentioning therefore, that recently Kulikova et al.. revealed that the Tyr52 residue of *Clostridioides difficile *Met15p (corresponding to Tyr54 in CaMet15p) is involved in ensuring the optimal position of the catalytic PLP-binding lysine residue at the stages of Cα proton abstraction^28^. Our results suggest that Tyr54 and Ala379 participate in the ligand/receptor complex stabilization via hydrogen bonds and amide/tyrosine stacking interactions (Fig. 9). Since in the substrate (OAH) structure, the amide bond is replaced with ester, both these stabilizing effects are lost, what seems to contribute to the observed difference in affinities of the two molecules to the enzyme.

As already mentioned, during the reaction catalyzed by Met15p, the substrate molecule forms a Schiff base with PLP. To check how much the covalent bond formation is going to influence the conformation of the bound ligand and in consequence its interaction pattern with the receptor, additional covalent docking calculations were performed. The results obtained for OAH and AcDAB are presented in Fig. 9A, B.

Comparing results of molecular docking of OAH (Fig. 9A) and AcDAB (Fig. 9B), one may observe that conformation of the bound ligands are nearly identical, except AcDAB being pulled deeper into the binding site, toward the PLP molecule, thus increasing the significance of the described interactions with Ala379 and Tyr54 (shortening the ligand-residue distances to 3.2Å and 3.7 Å respectively). At the same time, this shift allowed for some additional space inside that area of the binding site. These observations prompted us to design a structure of an AcDAB analog, namely L-4-benzamido-2-aminobutanoic acid (BzDAB), in which the acetamide is replaced with a bulkier benzamide hydrophobic group in order to enhance the interactions with this pocket and at the same time lipophilicity of the ligand, while retaining all other stabilizing effects. Results of docking of the newly proposed potential inhibitor BzDAB confirmed its improved ability to bind to the receptor in silico (Fig. 9C). Thus, we have decided to synthetize BzDAB (Figure S10) and analyze its ability to inhibit CaMet15NHp. BzDAB appeared to be active against CaMet15NHp although its inhibitory effectiveness turned out to be worse than expected (~ 44% of inhibition at 10 mM concentration) Concentration-dependent inhibitory potential of BzDAB is presented in Figure S12. Nevertheless, further attempts aimed at optimizing structure of a OAH analog as Met15p inhibitor seem worth trying.

### Inhibitors of CaMet15p demonstrate antifungal in vitro activity

The most active inhibitors of CaMet15p identified by us were tested for their antifungal in vitro activity *via* the serial two-fold dilution microplate method against *C. albicans*,* C. glabrata*, and *S. cerevisiae*. Since Met15p is involved in the L-methionine biosynthesis pathway, the Minimal Inhibitory Concentrations (MICs) were determined in the YNB minimal medium supplemented or not with 10 mM of L-Met. Ammonium sulfate (AS) or sodium glutamate (SG) were used as a nitrogen source in these determinations. The MIC values were also determined in the complex RPMI 1640 medium, composition of which mimics that of the low molecular weight compounds pool of human serum. The obtained results are given in Table 1.

**Table 1:** Susceptibility of yeast strains to L-4-acetamido-2-aminobutanoic acid (AcDAB), DL-2-allyglycine (DL-ALG), DL-glufosinate (DL-GLUF), Fluconazole (FLU). Minimal inhibitory concentration (MIC) is defined as the concentration of a compound at which fungal growth is inhibited by 50% (MIC_50_) or 90% (MIC_90_). Lack of any measurable activity of a given compound is represented by > 1024. The experiments were performed in triplicates.

Compound	Medium	MIC_90_ (MIC_50_) [µg∙mL^−1^]		
		*C. albicans* ATCC 10231	*C. glabrata* ATCC 90030	*S. cerevisiae* ATCC 9763
AcDAB	YNB AS	> 1024 (1024)	> 1024	> 1024
	YNB AS + L-Met	> 1024	> 1024	> 1024
	YNB SG	1024 (128)	> 1024 (512)	> 1024 (512)
	YNB SG + L-Met	> 1024	> 1024	> 1024
	RPMI 1640	> 1024	> 1024	> 1024
DL-GLUF	YNB AS	128 (16)	64 (16)	128 (8)
	YNB AS + L-Met	> 1024 (512)	1024 (128)	256 (32)
	YNB SG	256 (128)	128 (16)	128 (16)
	YNB SG + L-Met	> 1024 (1024)	1024 (32)	> 1024
	RPMI 1640	> 1024	> 1024	> 1024
DL-ALG	YNB AS	512 (16)	16 (4)	16 (4)
	YNB AS + L-Met	> 1024	> 1024	512 (128)
	YNB SG	64 (8)	16 (4)	8 (4)
	YNB SG + L-Met	> 1024 (256)	512 (64)	256 (32)
	RPMI 1640	> 1024 (1024)	> 1024	16 (4)
BzDAB	YNB AS	> 1024	> 1024	> 1024
	YNB AS + L-Met	> 1024	> 1024	> 1024
	YNB SG	> 1024	> 1024	> 1024
	YNB SG + L-Met	> 1024	> 1024	> 1024
	RPMI 1640	> 1024	> 1024	> 1024
FLU	RPMI 1640	4	32	8

It can be noted, that despite its high CaMet15p inhibitory activity, AcDAB exhibited very poor, if any, antifungal activity in all media tested, except for a slight effect observed in YNB SG medium. The antifungal effect of this compound was completely abolished, when the minimal medium was supplemented with L-Met, thus providing evidence for targeting the L-Met biosynthetic pathway. Antifungal activity of DL-ALG and DL-GLUF was better, in spite of their lower inhibitory potential against Met15p. One may expect even higher activity for pure L enantiomers of these compounds. The presence of L-Met in YNB media also abolished antifungal activity of these inhibitors, although in a few cases some remaining growth inhibitory effect was observed. This may suggest that both compounds have additional targets in yeast cells. DL-GLUF (also known as phosphinothricin) is a widely used herbicide that targets glutamine synthetase, the second most abundant protein in plant leaves, that is essential for nitrogen metabolism by catalyzing the adenosine triphosphate (ATP)-dependent incorporation of ammonia into L-glutamate to yield L-glutamine^33^. DL-GLUF is also able to inhibit this enzyme of the yeast *S. cerevisiae*in such a way that, although it is not used as a fungicide, it may alter amino acid biosynthesis in general^34^. On the other hand, L-ALG is well known as an inhibitor of glutamate decarboxylase^35^, an enzyme also present in yeast cells^36^. Antifungal effect of this compound has been already reported^37^. In our hands, DL-ALG was the only one of the Met15p inhibitors tested, that exhibited antifungal activity in RPMI 1640 medium against the *S. cerevisiae*, MIC_90_ value at the level of 16 µg mL^−1^.

A possible dual targeting of DL-GLUF and DL-ALG in yeasts may explain their better antifungal activity in comparison with AcDAB, targeting exclusively Met15p, but an inefficient transport of the latter to the cells cannot be excluded.

As previously reported, in the case of L-penicillamine (L-PEN) antifungal activity, *C. glabrata*, and *S. cerevisiae* growth in the minimal medium is more affected by this compound than that of *C. albicans*^9^. Since the same phenomenon has been observed now for DL-GLUF and DL-ALG, it is supposed to be related to the same mechanism. The reasoning behind these results may be the fact that either *C. glabrata* or *S. cerevisiae* are incapable of L-cysteine synthesizing *via* the *O*-acetyl-L-serine pathway^12,18^ (Fig. 2) which causes these species to be more vulnerable to inhibitors of Met15p or Met2p in minimal media. The generally lower activity of inhibitors tested in the minimal medium containing AS as a nitrogen source in comparison with that in the YNB SG medium, is most probably caused by the slower uptake of amino acid inhibitors to yeasts cells, due to the known phenomenon of inhibition of amino acids transport by ammonium ions^38^.

A synergistic growth inhibitory effect of compounds targeting two different enzymes of the same biosynthetic pathway is a known phenomenon, just to remind the antibacterial effect of sulfamethoxazole and trimethoprim in combination. Since Met2p and Met15p catalyze the two consecutive steps in the methionine biosynthetic pathway, a synergistic effect of their inhibitors seemed possible. The checkerboard microdilution method was used to study the interaction between L-penicillamine, targeting Met2p and Met15p inhibitors identified in this work. Detailed results of this analysis in the heatmap form are shown in Figure S11 and MIC_90_ and FICI (Fractional Inhibitory Concentration Index) parameters are presented in Table 2. Assuming that the drug interaction is considered synergistic if FICI ≤ 0.5, indifferent for 0.5 < FICI < 4 and antagonistic when FICI ≥ 4, a synergistic growth inhibitory effect was observed for combination of L-PEN with DL-ALG and L-PEN with AcDAB. These results indicate that simultaneous inhibition of Met2p and Met15p, i.e. enzymes catalyzing two initial consecutive steps of the methionine biosynthetic pathway may cause a strong synergistic effect, being a consequence of substantial lowering of MIC_90_ values of individual agents when in combination. It is noteworthy that these effects were more significant in the case of *C. glabrata*, i.e. yeasts lacking the *O*-acetyl-L-serine salvatory pathway of methionine biosynthesis.

On the other hand, DL-GLUF interaction with L-PEN was found indifferent. This finding seems to confirm that the primary target of DL-GLUF (also known as phosphinothricin) in *C. albicans* and *C. glabrata* is glutamine synthetase, and its inhibition of Met15p is of a secondary importance for growth inhibitory effect.

**Table 2:** The MIC_90_ values and FICI parameters resulting from the the checkerboard analysis of drug interaction between L-penicillamine (L-PEN) and L-4-acetamido-2-aminobutanoic acid (AcDAB), DL-2-allyglycine (DL-ALG) or DL-glufosinate (DL-GLUF) against *C. Glabrata* and *C. Albicans* in YNB minimal medium containing sodium glutamate (SG) as a nitrogen source. FICI—Fractional Inhibitory Concentration Index. *Value used for FICI calculations, although MIC_90_ was not actually reached.

Inhibitor	MIC_90_ [µg∙mL^−1^]		FICI	Effect
	Alone	In combination		
*C. glabrata* ATCC 90030				
AcDAB	2048*	128	0.313	Synergy
L-PEN	512	128		
DL-ALG	8	0.5	0.0938	Synergy
L-PEN	512	16		
DL-GLUF	128	64	1.00	Indifferent
L-PEN	512	256		
*C. albicans* ATCC 10231				
AcDAB	1024	128	0.375	Synergy
L-PEN	1024	256		
DL-ALG	64	16	0.50	Synergy
L-PEN	1024	256		
DL-GLUF	256	512	1.50	Indifferent
L-PEN	1024	1024		

## Conclusions

The major aim of the work was to identify the *Candida albicans* gene, coding *O*-acetyl-L-homoserine sulfhydrylase, and verify if that methionine biosynthesis pathway enzyme may be considered as an antifungal target. Cloned, expressed in *Escherichia coli*, and purified His-tagged fusion proteins showed the expected activity and were inhibited by synthetized L-4-acetamido-2-aminobutanoic acid and L-4-benzamido-2-aminobutanoic acid as well as commercially available compounds: namely DL-2-allyglycine, DL-glufosinate and L-penicillamine. Enzymatic studies showed a medium level of CaMet15p inhibition, but it turned out to be at a sufficient level to observe L-methionine-dependent antifungal activity. L-PEN appeared to be active as an inhibitor of both L-methionine biosynthesis pathway enzymes: Met15p as well and, as previously published, Met2p^9^. *C. glabrata* and *S. cerevisiae* growth in the minimal medium was more affected due to that strains inability to use two routes to produce L-Met: transsulfuration as well as the pathway related to L-Cys biosynthesis by cystathionine-γ-synthase (Str2p) and cystathionine-β-lyase (Str3p) (Fig. 1). Due to the fact that *S. cerevisiae* and *C. glabrata* possess only transsulfuration pathway that strains are more sensitive to Met15p inhibitors. Thus, one may conclude that Met15p targeting is not a universal approach in the search for antifungal compounds. However, our results, regarding the synergistic antifungal effects of the Met15p inhibitors (DL-ALG, AcDAB) and a previously published Met2p inhibitor (L-penicillamine), observed for both *C. albicans* and *C. glabrata* is a valuable discovery. Our results indicated that firstly, simultaneous inhibition of two pathway enzymes is possible and secondly, targeting the L-methionine biosynthetic pathway may be considered generally for all fungal cells, nevertheless of the presence or absence of both transsulfuration as well as the pathway related to L-Cys biosynthesis. Structures of L-penicillamine, L-4-acetamido-2-aminobutanoic acid, L-4-benzamido-2-aminobutanoic acid and DL-2-allyglycine may serve as a good starting point for the development of novel inhibitors with higher inhibitory rates.

## Methods

### Reagents

The commercially available reagents, namely L-homocysteine; *O*-acetyl-L-serine; sodium sulfide nonahydrate; pyridoxal phosphate (PLP); guanidinium-HCl, 5,5’-dithio-bis(2-nitrobenzoic acid) (DTNB); acetyl-L-carnitine; L-penicillamine; 3-(2-thienyl)-L-alanine; L-methionine sulfoximine; DL-phosphinothricin; L-2,4-diaminobutyric acid dihydrochloride, DL-2-allylglycine)were purchased from Sigma-Aldrich, USA.

### Synthesis of OAH and AcDAB

The scheme of OAH as well as AcDAB synthesis is presented in Figure S7.

**Synthesis of *N***^***γ***^**-acetyl-l−2**,**4-diaminobutanoic acid (Figure S7.3)** starts with a reaction between Cbz-L-glutamine and bis(trifluoroacetoxy)iodobenzene (PIDA), resulting in an appropriately protected l−2,4-diaminobutanoic acid (Figure S7.1)^39^. Treatment with an acetic anhydride in the presence of pyridine gives *N*^*γ*^-acetyl product (Figure S6.2)^40^. Final catalytic hydrogenolysis leads to the final compound (Figure S7.3).

*O*-acetyl-l-homoserine **4**is obtained in two subsequent steps^41^. First, l-homoserine is treated with perchloric acid and acetic anhydride in the presence of acetic acid to form intermediate cyclic products. Then, intermediate products are decomposed by the treatment of *n*-butylamine and the final product is isolated from the reaction mixture^41^.

***N*-Benzyloxycarbonyl-(*S*)−2**,**4-diaminobutanoic acid (Figure S7.1)**. To the solution of N-benzyloxycarbonyl-(*S*)-glutamine (1 g, 3.57 mmol) in THF (10 mL) and water (2.5 mL) bis(trifluoroacetoxy)iodobenzene PIDA (1.36 g, 4.29 mmol) was added in one portion. The resulting mixture was stirred magnetically at 4 ^o^C for 8 h. Then, solvents were evaporated and the resulting foamy solid was dissolved in water (10 mL) and extracted with ethyl acetate (3 × 10 mL). The aqueous layer was evaporated yielding a crude product, which was washed with a mixture of ethyl acetate-chloroform (1:1, v/v, 20 mL) to afford **2** (710 mg, 79%) as a white solid. [$\:\alpha\:$]_D_ = −9.6 (*c*1, H_2_O)^1^H NMR (500 MHz, DMSO-d_6_): δ = 1.69 (m, 1 H, *CH*_*2*_CH), 1.88 (m, 1 H, *CH*_*2*_CH), 2.82–2.91 (m, 2 H, NH_2_*CH*_*2*_), 3.6 (m, 1 H, CH_2_*CH*), 4.99 (s, 2 H, *CH*_*2*_C_6_H_5_), 6.59 (d, 1 H, *NH*Z), 7.24–7.39 (m, 5 H, *C*_*6*_*H*_*5*_).

***N***^4^***-*acetyl*-N***^2^**-Benzyloxycarbonyl-(*S*)−2**,**4-diaminobutanoic acid (Figure S7.2).** The *N*-Benzyloxycarbonyl-(*S*)−2,4-diaminobutanoic acid (500 mg, 1.98 mmol) **2** was dissolved in dry pyridine (5 mL) and acetic anhydride (0.188 mL, 1.98 mmol) was added. After stirring at room temperature for 1 day, the mixture was acidified to pH 1 with 1 M HCl. The resulting solution was extracted with ethyl acetate (3 × 10 mL) and combined organic layers were extracted with 5% NaHCO_3_ (3 × 10 mL). The water layer was acidified and extracted with ethyl acetate (3 × 10 mL). Organic layers were polled and then washed with saturated brine and dried over Na_2_SO_4_. After evaporation of solvent crude product was obtained, which was purified by column chromatography using chloroform/methanol/ acetic acid (65:10:1) as eluent. Product as a white solid (350 mg, 60%)^1^. H NMR (500 MHz, D_2_O): δ = 1.84–1.92 (m, 2 H, *CH*_*2*_CH), 1.94 (s, 3 H *CH*_*3*_CO), 3.27 (t, 2 H, *CH*_*2*_NH), 4.2 (m, 1 H, *CH*CH_2_), 5.13 (s, 2 H, *CH*_*2*_C_6_H_5_), 7.36–7.49 (m, 5 H, *C*_*6*_*H*_*5*_).

***N***^4^***-*acetyl-(*S*)−2**,**4-diaminobutanoic acid (Figure S7.3). ***N*^4^*-*acetyl*-N*^2^-Benzyloxycarbonyl-(*S*)−2,4-diaminobutanoic acid (350 mg, 1,19 mmol) was dissolved in 5 mL of THF and 10 mg of 10% palladium catalyst Pd/C was added. The mixture was stirred under H_2_ for 4 h. Reaction was filtrated and concentrated *in vacuo* resulting in pure product as a white solid (187 mg, 98%). [$\:\alpha\:$]_D_ = −10.7 (*c*1.4, EtOH)^1^, H NMR (500 MHz, D_2_O): δ = 1.96 (s, 3 H, *CH*_*3*_CO), 1.97–2.12 (m, 2 H, *CH*_*2*_CH), 3.22–3.28 (m, 1 H *CH*_*2*_NH), 3.31–3.37 (m, 1 H, *CH*_*2*_NH), 3.68 (dd, 1 H, *CH*CH_2,_J = 7.6, 5.6 Hz)^13^, C NMR (500 Hz, D_2_O): δ = 21.6 (*CH*_*3*_CO), 30.0 (*CH*_*2*_CH), 35.3 (*CH*_*2*_NH), 52.3 (*CH*CH_2_), 174.1 (*COOH*), 174.6 (CH_3_*CO*), MS ESI^+^ : 161 (M + H^+^, observed), 144 (M-NH_2,_ observed), 183 (M + Na, observed) 160 (M, calculated), MS ESI^−^ : 159 (M-H^+^, observed), 160 (M, calculated) (Figure S9).

***O-*acetyl-(*S*)-homoserine (Fig. 7.4).** To a glacial acetic acid (2 mL) 0.2 mL of perchloric acid (60%) was slowly added. Then, the mixture was slightly cooled and 1 mL of acetic anhydride was added to the solution. 150 mg of L-homoserine (1.26 mmol) dissolved in 1 mL of glacial acetic acid was added dropwise to the solution. The reaction was stirred for 2 h at room temperature. Then, 0.05 mL water was added followed by 0.3 mL of *n*-butylamine. The reaction mixture was poured into 30 mL of cold diethyl ether and left at −20 ^o^C for crystallization. After several hours precipitate was collected and dissolved in 1.5 mL of water. 10 mL of ethanol was added, and the solution was kept at 4 ^o^C to crystallization overnight. The product was obtained through filtration as pearly thin plates with a strong shine (120 mg, 59%)^1^. H NMR (500 MHz, D_2_O): δ = 2.08 (s, 3 H, *CH*_*3*_NO), 2.14–2.22 (m, 1 H, *CH*_*2*_CH), 2.22–2.30 (m, 1 H *CH*_*2*_CH), 3.82 (dd, 1 H, CH_2_*CH*, J = 7.1, 5.3 Hz), 4.22 (t, 2 H, O*CH*_*2*,_J = 6.0 Hz)^13^C NMR (500 Hz, D_2_O): δ = 20.2 (*CH*_*3*_CO), 29.1 (*CH*_*2*_CH), 52.6 (*CH*CH_2_), 61.4 (*CH*_*2*_O), 173.8 (*COOH*), 174.0 (CH_3_*CO*), MS ESI^+^ : 162 (M + H^+^, observed), 184 (M + Na, observed) 161 (M, calculated). (Figure S8).

### Synthesis of BzDAB

Synthesis of 3-benzamido-1-carboxypropan-1-aminium chloride (Figure S10.3) starts from commercially available BOC-protected L-2,4-diaminobutanoic acid **1**, which is treated with benzoyl chloride under alkaline conditions. Resulting compound **2** after deprotection was converted into chloride salt to enhance solubility of the obtained product **3**.

**4-benzamido-2-((tert-butoxycarbonyl)amino)butanoic acid (Figure S10.2)**. Commercially available BOC-protected L-2,4-diaminobutanoic acid **1** (1 g, 4,79 mmol) and NaOH (390 mg, 9,63 mmol) were dissolved in dioxane/water (1: 1, 0.5 M). The solution was cooled in an ice bath, and benzoyl chloride (670 µl, 5,75 mmol) was added dropwise. Reaction mixture was stirred overnight at RT. Acidified the cooled reaction mixture with 3 M aq. HCl. Extracted products with diethyl ether, dried (MgSO4), and concentrated in vacuo. Crude products were purified by column chromatography using hexane/ethyl acetate/acetic acid (60:40:1) as eluent. Product was obtained as a white solid (1,24 g, 84% yield). 1 H NMR (500 MHz, DMSO): δ = 1.36 (s, 9 H, (CH3)3 C), 1.79 (m, 1 H, CH2-CH), 1.97 (m, 1 H, CH2-CH), 3.27–3.32 (m, 2 H, CH2-NH), 3.97 (m, 1 H, CH-CH2), 7.14–3.47 (m, 5 H, C6H5), 12.5 (bs, 1 H, COOH); ). 13 C NMR (500 MHz, DMSO): δ = 28,64 (3 C, (CH3)3 C), 30,97 (1 C, CH2-CH2NH), 36,88 (1 C, CH2-NH), 51,89 (1 C, CH2-CH-HN), 78,49 (1 C, C(CH3)3), 127,59 (2 C, C6H5), 128,65 (2 C, C6H5), 131,5 (1 C, C6H5), 134,94 (1 C, C6H5), 156,00 (1 C, COOC(CH3)3), 166,68 (COC6H5), 174,43 (COOH).

**L-4-benzamido-2-aminobutanoic acid chloride (Figure S10.3).** 4-benzamido-2-((tert-butoxycarbonyl)amino)butanoic acid **2** (1 g, 3,11 mmol) was dissolved in 3 ml of DCM and 1 ml of THF was added to the solution. The mixture was stirred at RT for 2 h. After completion of the reaction, solvents were evaporated, and diethyl ether was added resulting in white solid. Product was filtered and washed several times with diethyl ether. White solids were converted into a chloride salt using HCL/dioxane mixture. Obtained 0,74 g (2,86 mmol, 92% yield) of white solid. ).[α]D20 = + 3 (c 1, H2O) 1 H NMR (500 MHz, D2O): 2,00 (m, 2 H, CH2-CH), 3,30 (m, 2 H, CH2-NH), 3,87 (t, 1 H, CH-CH2), 7,18 − 7,44 (m, 5 H, C6H5). 13 C NMR (500 MHz, DMSO): 27,74 (1 C, CH2-CH), 30,8 (1 C, CH¬2-NH), 36,8 (1 C, CH-CH2), 127,68 (2 C, C6H5), 129,02 (2 C, C6H5), 132,02 (1 C, C6H5), 135,03 (1 C, C6H5), 166,8 (1 C, COPh), 170,82 (COOH). MS ESI+: 223 (M + H+, observed).

### Strains and plasmids

*Escherichia coli* One Shot™ TOP10 cells (Invitrogen, MA, USA) were used in the cloning procedures. *Escherichia coli* ER2566 (New England Biolabs, MA, USA) was used for expression with plasmids based on the LIC cloning and expression system with pLate52 expression vector (Thermo Scientific, MA, USA). The antimicrobial activity of chosen inhibitors was tested on *Candida albicans* ATCC 10231, *Candida glabrata* ATCC 90030, and *Saccharomyces cerevisiae* ATCC 9763. Bacterial strains were cultured on solid (1.5% m/V agar) and liquid Luria-Bertani (LB) media (0.5% yeast extract; 1.0% peptone; 1.0% NaCl; ampicillin 100 µg mL^−1^). Yeast strains were cultured on solid and liquid YPG medium (1% yeast extract; 1% peptone; 2% glucose; 1.5% agar).

### Bioinformatical analysis

The Met15p encoding gene sequence was retrieved from the *Candida *Genome Database^19^. The amino acid sequence and the molecular weight of the expected gene product were retrieved by the ProtParam tool^20^. The multiple sequence alignment was conducted by the BLAST tool^42^. Rendering of aligned sequence similarities was performed with ESPript 3.x software^43^.

### Molecular modeling

The structure of the Met15p receptor for docking calculations was prepared based on the AlphaFold^31^ model and the structure of the enzyme from *S. cerevisiae* (8OVH). The dimeric form of the protein was used as the receptor since the single binding site is formed by residues belonging to the two distinct chains. The placement and conformation of the PLP molecule present in the binding site were also modeled based on its conformation in the *S. cerevisiae* structure and it was fixed as part of the receptor during all docking simulations.

All ligands were first built using HyperChem software [HyperChem(TM) Professional 8.0, Hypercube, Inc., 1115 NW 4th Street, Gainesville, Florida 32601, USA], and then converted to pdbqt format with AutoDockTools scripts^44^. The docking grid was centered on the PLP aldehyde moiety, and its size was set to 35 A in each direction. 50 docking simulations were performed for each ligand using Autodock 4.2 and LGA algorithm^44^. The results were clustered and low-energy poses representing the most abundant clusters were selected for analysis.

Since the reaction catalyzed by the enzyme involves the formation of a Schiff base to the PLP molecule, besides the standard semi-flexible docking protocol, additional covalent docking was performed for each ligand, with its α-amino moiety bound to the PLP. To achieve this additional interaction map was created with a Gaussian potential centered on the position of the bound nitrogen with width δ and amplitude ε parameters set to 3Å and 10 kcal/mol respectively.

All figures presenting the results of molecular modeling were prepared with VMD software^45^.

### Met15 cloning and expression

The Met15 sequence corresponding to the orf19.5645 was amplified using the *Candida albicans* SC5314 genome as a matrix. Primers used for the amplification of the 6xHis-tagged Met15NHp gene were 5’-GGTTGGGAATTGCAACCTTCTCACTTTG-3’ and 5’-GGAGATGGGAAGTCATTAGTTGTTATAAACCTT-3’ as forward and reverse primers respectively. Primers used for the amplification of the 6xHis-tagged Met15CHp gene were 5’-AGAAGGAGATATAACTATGCCTTCTCACTTTGATACTCTTCAATT-3’ and 5’-**GTGATGATGATGATGATG**GCCG TTGTTATAAACCTTCT-3’ as a forward and a reverse primer respectively. Obtained PCR products were cloned into pLate52 and pLate31 vectors, respectively for the NH-his-tagged and CH-his-tagged variants, using an aLICator Ligation Independent Cloning and Expression System (Thermo Scientific, MA, USA). Obtained plasmids were verified by nucleotide sequencing. *Escherichia coli* ER2566 cells transformed with pLate31:Met15CH or pLate52:Met15NH were inoculated into LB broth supplemented with 100 µg mL^−1^ ampicillin and left to grow overnight at 30 °C. 10 mL of the starter culture was added to 800 mL LB broth supplemented with ampicillin and incubated at 30 °C (200 rpm) until the OD_600_ reached 1.0. 1 mM of isopropyl-β-D-thiogalactoside (IPTG) was added to induce protein expression, after which the cells were incubated for 24 h at 15 °C (200 rpm) before cell harvesting.

### Protein purification

The cell pellet was resuspended in buffer A (20 mM Tris-HCl buffer pH 8.0; 5 mM imidazole; 500 mM NaCl; 1 mM Tween 20) supplemented with cOmplete™ Protease Inhibitor Cocktail (Hoffmann-La Roche, Switzerland); 10 mM dithiothreitol (DTT)) and sonicated on ice following centrifugation at 10.000 rpm for 20 min. The resulting supernatant was subjected to affinity chromatography with a 5 mL HisTrap™ Fast Flow (Cytiva, MA, USA) column in a ӒKTA Pure™ chromatography system (Cytiva, MA, USA). The column was equilibrated with buffer A before sample injection, and the target protein was eluted with buffer B (20 mM Tris-HCl buffer pH 8.0; 500 mM imidazole; 500 mM NaCl; 1 mM Tween 20) with a purity rate of 93% showed by an SDS-PAGE analysis. Gels were imaged using the Gel Doc XR + Gel Documentation System (Bio-Rad: Hercules, CA, USA) to take photographs.

### Enzymatic activity measurements

The amount of L-homocysteine (HCT) produced was measured by a HPLC-DAD-MS method with a previous derivatization of L-HCT with Ellman’s reagent. The reaction mixture contained 50 mM Tris-HCl pH 8.0; 100 mM NaCl; 0–100 mM *O*-acetyl-L-homoserine; 0–3 mM Na_2_S; and 0.2 mM PLP. The reaction was started by the addition of 10 nM pure enzyme and run for 10 min at 37 °C. The reaction was terminated by the addition of a stopping buffer (100 mM sodium phosphate pH 5.8; 3.2 mM guanidinium-HCl) followed by the addition of 3 mM Ellman’s reagent. Samples were injected into HPLC-DAD (Zorbax Eclipse C18 column 5 μm, 250 mm x 4.6 mm (Agilent, CA, USA); mobile phase A:0.45% HCOOH, B: ACN. 1.2 ml min ^−1^). HCT was detected at λ = 330 nm. Inhibitors were dissolved in H_2_O and added to the reaction mixture prior to the addition of an enzyme to final concentrations of 2 mM, 5 mM, and 10 mM. Kinetic parameters were calculated with GraphPad Prism 8.0 (GraphPad Software Inc., CA, USA). All measurements were performed in triplicate.

### Antifungal in vitro activity determination

Yeasts cells were cultured on YPG plates overnight. The picked colonies were used as inoculum for Minimal Inhibitory Concentration (MIC) assay, performed in RPMI-1640 medium, according to a M27-A3 procedure specified by the CLSI^46^. MICs determination in minimal YNB media was performed according to the modified M27-A3 protocol. Briefly, fungal strains were first cultivated on YPG agar plates for 24 h at 30 °C. Overnight cultures were then suspended in phosphate-buffered saline to reach an optical density of 0.1 measured at 600 nm. Serial dilutions of selected inhibitors and fluconazole (Sigma-Aldrich) were inoculated with culture of tested fungal strains obtaining a final concentration of ~ 10^4^ colony forming units (CFU) per mL medium in 96-well plates. Tested mediums include the Yeast Nitrogen Base (YNB) medium with ammonium sulfate (AS) or sodium glutamate (SG) as a nitrogen source, and with or without supplementation of 10 mM L-methionine. The assay was conducted in 96-well flat bottom plates that were next incubated at 37 °C for 24 h. The growth rate of strains was measured via the optical density at 600 nm with a microplate reader (TECAN Spark 10 M, Austria). The MIC_90_ and MIC_50_ parameters are defined as the lowest concentration of antifungal compound that inhibited growth by at least 90% or 50%, respectively. All measurements were performed in triplicate.

### Oligomeric structure and molecular mass determination

Bioinformatical analysis was performed with the help of the ProtParam^20^and Translate^47^ programs and the nucleotide sequence of Met15p retrieved from the *Candida *Genome Database^19^.

Size exclusion chromatography (SEC): 0.5 mL of concentrated 1 mg mL^−1^ protein solution was loaded onto Superdex^®^ 200 10/300 GL increase (Cytiva, MA, USA) column. The elution was carried out in a sodium phosphate elution buffer in 1.5 column volumes. 0.5 mL fractions were collected and examined with the help of SDS-PAGE electrophoresis. Fractions containing the analyzed protein were stored for later use at protein-optimal conditions. To determine the molecular mass, size exclusion chromatography of reference proteins was conducted using Gel Filtration Markers Kit for Protein Molecular Weights 29,000–700,000 Da (Sigma-Aldrich, MO, USA) according to a given protocol. A standard curve showing the relationship between molecular mass and volume of elution of protein was made. 0.5 mL of concentrated 1 mg mL^−1^ protein solution was loaded onto the Superdex^®^ 200 10/300 GL increase column (Cytiva, MA, USA). Solutions: SEC elution buffer: 50 mM Tris-HCl buffer pH 8; 300 mM NaCl.

Native-PAGE electrophoresis was carried out with the help of the NativePAGE™ Novex Bis-Tris Gel System kit (Invitrogen, MA, USA) and according to the given procedure. Samples were loaded on 4–16% bis-Tris-HCl gels. Staining of gels was made using the NativePAGE™ Novex Bis-Tris Gel System kit (Invitrogen, MA, USA) method. Mass standard used: Native MARK™ Unstained Protein Standard (Thermo Scientific, MA, USA). The assessment of the molecular mass of bands appearing on the polyacrylamide gels was performed using the Gel Analyzer (version 19.1) program^48^.

### Western blotting analysis

Electroblotting was performed for 1 h at 40 mA using a semi-dry Blot transfer. After the transfer of proteins, the nitrocellulose membrane was placed in a 5% skim milk solution overnight. The nitrocellulose membrane was washed 3 times with 1:10 wash buffer (10 mM Tris-HCl, pH 8.0, 30 mM NaCl) for 30 min, followed by 1-hour incubation in a solution containing Anti-polyHistidine − Peroxidase antibody, Mouse monoclonal (Sigma-Aldrich, MO, USA). The membrane was then washed 3 times with a 1:10 wash buffer for 60 min. The staining of bands containing 6xHis-tag was made with 1 mL of 3,3′,5,5′-Tetramethylbenzidine Liquid Substrate System for Membranes (Sigma-Aldrich, MO, USA). Blots were imaged using the Gel Doc XR + Gel Documentation System (Bio-Rad: Hercules, CA, USA) to take photographs.

### Checkerboard dilution test for determination of drug interaction

Potential synergistic, antagonistic, or indifferent effects were tested for interaction between L-penicillamine (L-PEN) and L-4-acetamido-2-aminobutanoic acid (AcDAB), DL-2-allyglycine (DL-ALG) or DL-glufosinate (DL-GLUF). Interactions were checked in a minimal medium (YNB) supplemented with sodium glutamate (SG) as the sole nitrogen source. Two-fold dilutions of inhibitors (AcDAB, DL-ALG, or DL-GLUF) were spread along the y-axis of the 96-well flat bottom plate followed by the distribution of two-fold dilution of L-PEN along the x-axis. Final concentrations of tested compounds range from at least 2 times MIC to 1/32 MIC. The prepared 96-well plate was next inoculated with a volume fungal inoculum equal to the volume of diluted tested compounds resulting in the final concentration of cells ~ 10^4^ colony-forming units (CFU) mL^−1^ medium. Plates were incubated at 37 °C for 24 h. The growth rate of strains was measured via the optical density at 600 nm with a microplate reader (TECAN Spark 10 M, Austria). The MIC_90_ parameters are defined as the lowest concentration of antifungal compound that inhibited growth by at least 90%. All measurements were performed in triplicate. The fractional inhibitory concentration index (FICI) was calculated according to the below formula:

$$\:\text{FICI}\text{=}\frac{\text{MIC}\text{ }\text{of}\text{ }\text{L}\text{-}\text{PEN}\text{ }\text{in}\text{ }\text{co}\text{m}\text{bination}}{\text{MIC}\text{ }\text{of}\text{ }\text{L}\text{-}\text{PEN}\text{ }\text{alone}}\text{+}\frac{\text{MIC}\text{ }\text{of}\text{ }\text{inhibitor}\text{ }\text{in}\text{ }\text{combination}}{\text{MIC}\text{ }\text{of}\text{ }\text{inhibitor}\text{ }\text{alone}}$$

Calculated FICIs were interpreted according to the instruction given by Odds^49^, where synergy is defined by FICI ≤ 0.5, indifferent effect (FICI > 0.5 do ≤ 4), and antagonistic (FICI > 4). The synergy score was also visualized and analyzed with the help of Combenefit software (version 2.021, Cancer Research UK Cambridge Institute, UK)^50^ using the Loewe additivity model.

### Statistics and reproducibility

All experiments were carried out in triplicates, in two independent experimental sets. The means ± standard deviation (± SD) were used in the statistical analysis of the data and the graphics.

## Electronic supplementary material

Below is the link to the electronic supplementary material.

Supplementary Material 1

## Data Availability

Data generated or analyzed in this study are included in this published article and supplementary material files. Raw datasupporting our results are also available as data sets: (1) Gabriel, I., Kuplińska, A., Rząd, K., & Milewski, S. (2022).Identification and cloning of C. albicans SC5314 genes encoding L-methionine biosynthetic pathway enzymes. (1–) [dataset]. Gdańsk University of Technology. 10.34808/zac2-tz70; (2) Gabriel, I., Kuplińska, A., Kozłowska-Tylingo, K., & Rząd, K. (2023). Activity assay of O-Acetyl-L-homoserine sulfhydrylase (CaMet15p). (1–) [dataset]. Gdańsk University of Technology. 10.34808/4ta6-e594; (3) Gabriel, I., Kuplińska, A., & Rząd, K. (2023). Overproduction of CaMet15p native and His-tag versions. (1–) [dataset]. Gdańsk University of Technology. 10.34808/y9xa-5w80.
