# Supplementary material for: Targeting Candida albicans O-acetyl-L-homoserine sulfhydrylase (Met15p) in antifungal treatment
Source: Sci Rep. 2024 Nov 15;14:28188. doi: 10.1038/s41598-024-79886-y (PMC11568248; doi:10.1038/s41598-024-79886-y)
Supplement: Supplementary file 1 — Supplementary Material 1 [file 41598_2024_79886_MOESM1_ESM.pdf]

Supplementary data

Article

Targeting *Candida albicans* O-acetyl-L-homoserine sulfhydrylase (Met15p) in antifungal treatment.

Aleksandra Kuplińska [1], Kamila Rząd[1], Joanna Stefaniak -Skorupa[2], Katarzyna Kozłowska-Tylingo[1], Marek Wojciechowski[1], Sławomir Milewski[1] and Iwona Gabriel*[1]

[1] *Department of Pharmaceutical Technology and Biochemistry, Faculty of Chemistry, Gdansk University of Technology, 11/12 Narutowicza Str., 80-233 Gdansk, Poland*

[2] Department of Organic Chemistry, Gdansk University of Technology, Gdansk, Poland
*Correspondence: Tel: +48 583486078; E-mail: iwona.gabriel@pg.edu.pl

Table of contents:

No.	Name	Details
1	**Figure S1**	Sequence alignment of the amino acid sequence of the Met15p from *Candida albicans* (CA) with homologous sequences of characterized 3D structures of O-acetyl-L-homoserine sulfhydrylase enzymes. Analysis performed with Escript 3.0 software [1]. Conserved amino acid residues are shown in red boxes, The putative conserved catalytic residues of *C. albicans* Met15p are shown marked in black boxes with asterisk: Tyr54, Arg56, Gly84, Glu152, Asp181, Thr183, Ser204, Thr206, Lys207, Arg415. Other putative catalytic resides are marked with black circle. PDB codes: *S. cerevisiae* 8OVH, *Wolinella succinogenes* 3RI6, *Mycobacterium marinum* 4KAM.
2	**Figure S2**	Purification of Met15NHp and Met15CHp. A Result of SDS-PAGE gel electrophoresis of cell-free extract from E. *coli* ER2566 cells (CFE) producing Met15NHp enzyme. Met15NHp enzyme purified with FPLC and His-Trap FF 5mL column (Cytiva, Marlborough, MA, USA) (Met15NHp). B Densitometric analysis of SDS-PAGE electrophoresis of cell-free extract producing Met15NHp; C Densitometric analysis of SDS-PAGE electrophoresis of Met15NHp. D Result of SDS-PAGE gel electrophoresis of cell-free extract from *E. coli* ER2566 cells (CFE) producing Met15CHp enzyme, Met15CHp enzyme purified with FPLC and His-Trap FF 5mL column (Cytiva, Marlborough, MA, USA). (Met15CHp). E Densitometric analysis of SDS-PAGE electrophoresis of cell-free extract producing Met15CHp; F Densitometric analysis of SDS-PAGE electrophoresis of Met15CHp. Electrophoresis was performed at 18 V cm-1 10% gel. Thermo Scientific PageRuler™ Plus Prestained Protein Ladder (M) (Thermo Scientific, MA, USA). Densitometric analysis performed with Gel Analyzer 19.1 software[2]
3	**Figure S3**	Examples of chromatograms showing peak area of L-HCT-TNB; L-HCT produced in the reaction catalyzed by CaMet15NHp. The reaction mixture consists of 10 nM of enzyme, 0.2 mM PLP, 0.1 mM Na2S, and varying concentrations of L-OAH: A. 0 mM; B. 20 mM; C. 50 mM; D. 100 mM. Performed via Agilent 1200 Quaternary DAD HPLC System.
4	**Table S1**	The change in the surface area under the peak corresponding to HCT-TNB that was observed, depending on the concentration of the OAH substrate.
5	**Figure S4**	Kinetic parameters of Met15NHp determined for 10 nM enzyme at reaction set for 10 minutes in Tris-HCl pH 8.0 buffer. A K_m and V_{max} were determined for Na_2S at a constant concentration of OAH and PLP equal to 10 mM and 0.2 mM, respectively. B K_m and V_{max} determined for OAH at a constant concentration of Na_2S and PLP equal to 0.1 mM and 0.2 mM, respectively. Calculations were performed with GraphPad Prism 8.0.1 software.
6	**Figure S5**	Examination of storage conditions on the activity of Met15NHp. Measurement of the fresh enzyme was performed immediately after purification, after which an enzyme was stored for 22 hours at 4°C or 22°C, with or without supplementation of 20% glycerol.
7	**Figure S6**	Assessment of Met15NHp molecular mass and oligomeric structure by SDS-PAGE and NATIVE-PAGE electrophoresis. M, marker; AC, affinity chromatography; SEC, size exclusion chromatography. SDS-PAGE electrophoresis was performed at 18 V cm-1 10% gel. Thermo Scientific PageRuler™ Plus Prestained Protein Ladder (M) (Thermo Scientific, MA, USA). NATIVE-PAGE electrophoresis performed at 15 V cm^{-1}; NativePAGE™ Novex Bis-Tris Gel System kit (Invitrogen, MA, USA) 4-16% gel. Thermo Scientific Native MARK™ Unstained Protein Standard (M) (Thermo Scientific, MA, USA). Met15NHp was purified with

		HisTrap™ Fast Flow 5 mL column and a Superdex 13/300 GL increase column (Cytiva, MA, USA).
**8**	**Figure S7**	Synthesis of N$^{\gamma}$-acetyl-L-2,4-diaminobutanoic acid **3** and *O*-acetyl-L-homoserine **4**. Synthesis of N$^{\gamma}$-acetyl-L-2,4-diaminobutanoic acid **3** starts with a reaction between Cbz-L-glutamine and bis(trifluoroacetoxy)iodobenzene (PIDA), resulting in an appropriately protected L-2,4-diaminobutanoic acid **1**[3]. Treatment with an acetic anhydride in the presence of pyridine gives N$^{\gamma}$-acetyl product **2**[4]. Final catalytic hydrogenolysis lead to the final compound **3**. *O*-acetyl-L-homoserine **4** is obtained in two subsequent steps[5]. First, L-homoserine is treated with perchloric acid and acetic anhydride in the presence of acetic acid to form intermediate cyclic products. Then, intermediate products are decomposed by the treatment of n-butylamine and the final product is isolated from the reaction mixture[5].
**9**	**Figure S8**	ESI-MS spectrum for *O*-acetyl-L-homoserine.
**10**	**Figure S9**	ESI-MS spectrum for N$^{\gamma}$-acetyl-L-2,4-diaminobutanoic acid.
**11**	**Figure S10**	Synthesis of L-4-benzamido-2-aminobutanoic acid chloride (BzDAB) **3**
**12**	**Figure S11**	The result of the checkerboard assay of antifungal activity of combinations of L-penicillamine (L-PEN) with L-4-acetamido-2-aminobutanoic acid (AcDAB), DL-2-allyglycine (DL-ALG) or DL-glufosinate (DL-GLUF) in YNB-SG minimal medium against *C. glabrata* ATCC 90030. Combinatory effect scores calculations were obtained according to the Loewe additivity model via Combenefit software26. In Figs A, C and E the results are shown in the form of a heatmap representing the level of synergy at each concentration where synergy (a positive score) is shown in blue color, antagonism (a negative score) is shown in red color and an indifferent effect (score 0) is shown in green color. In Figs. B, D and F, the results are presented as percentages of growth compared to that of the untreated control.
**13**	**Figure S12**	Concentration-dependent inhibitory potential of BzDAB.
**14**	**Figure S13**	Original and uncut images from Figure 3. Left A; Right B.
**15**	**Figure S14**	Original and uncut gels from Figure S2. Left A, B, and C; Right D, E and F.
**16**	**Figure S15**	Original and uncut gels from Figure S6. Left SDS-PAGE; Right NATIVE-PAGE.

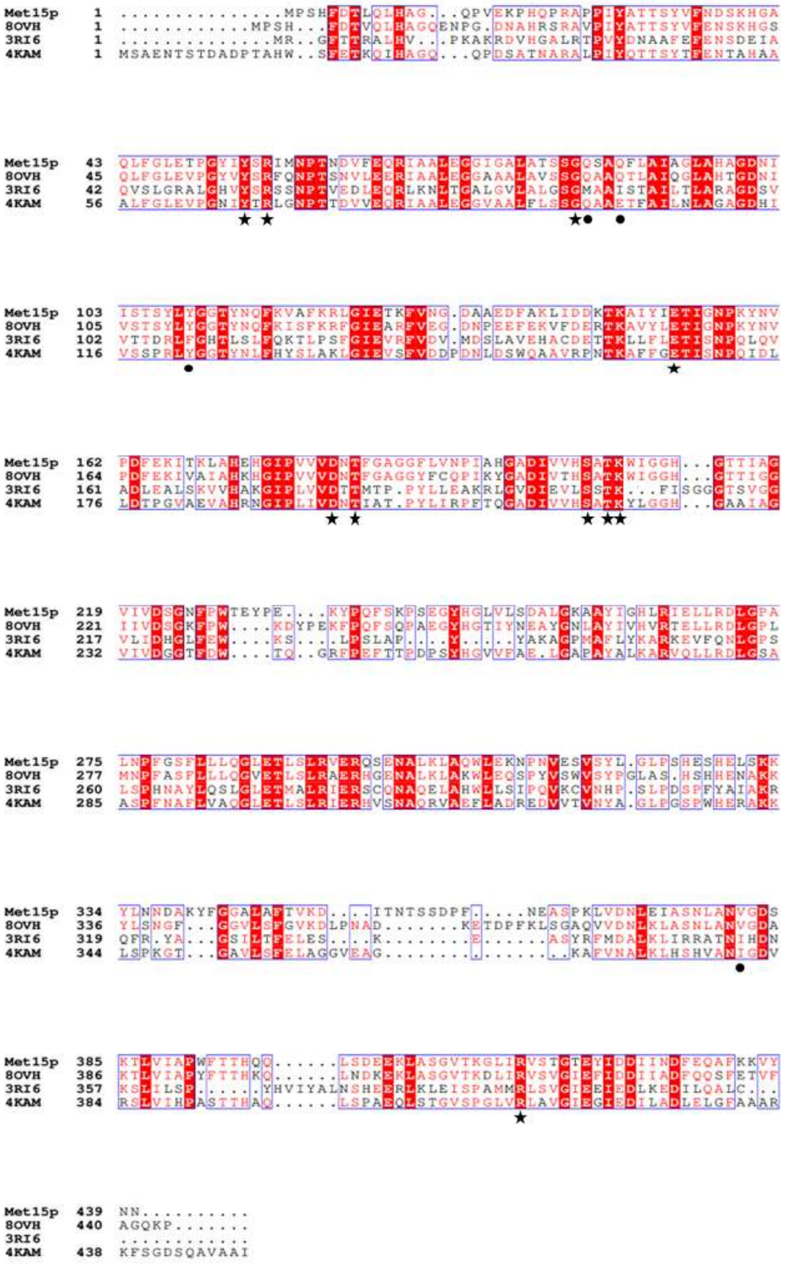

Figure S1. Sequence alignment of the amino acid sequence of the Met15p from *Candida albicans* (CA) with homologous sequences of characterized 3D structures of *O*-acetyl-L-homoserine sulfhydrylase enzymes. Analysis performed with Escript 3.0 software [1]. Conserved amino acid residues are shown in red boxes, The putative conserved catalytic residues of *C. albicans* Met15p are marked with asterisk: Tyr54, Arg56, Gly84, Glu152, Asp181, Thr183, Ser204, Thr206, Lys207, Arg415. Other putative catalytic resides are marked with black circle. PDB codes: *S. cerevisiae* 8OVH, *Wolinella succinogenes* 3RI6, *Mycobacterium marinum* 4KAM.

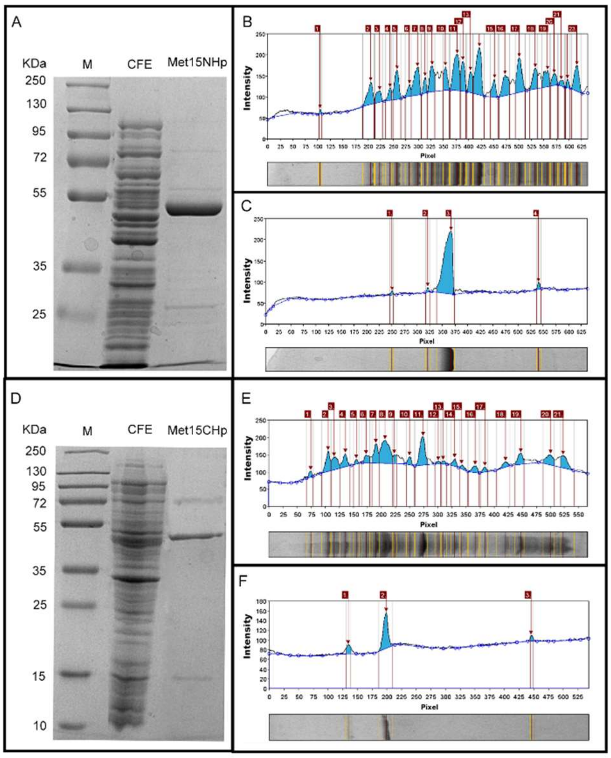

Figure S2. Purification of Met15NHp and Met15CHp. A Result of SDS-PAGE gel electrophoresis of cell-free extract from E. *coli* ER2566 cells (CFE) producing Met15NHp enzyme. Met15NHp enzyme purified with FPLC and His-Trap FF 5mL column (Cytiva, Marlborough, MA, USA) (Met15NHp). B Densitometric analysis of SDS-PAGE electrophoresis of cell-free extract producing Met15NHp; C Densitometric analysis of SDS-PAGE electrophoresis of Met15NHp. D Result of SDS-PAGE gel electrophoresis of cell-free extract from *E. coli* ER2566 cells (CFE) producing Met15CHp enzyme, Met15CHp enzyme purified with FPLC and His-Trap FF 5mL column (Cytiva, Marlborough, MA, USA). (Met15CHp). E Densitometric analysis of SDS-PAGE electrophoresis of cell-free extract producing Met15CHp; F Densitometric analysis of SDS-PAGE electrophoresis of Met15CHp. Electrophoresis was performed at 18 V cm-1 10% gel. Thermo Scientific PageRuler™ Plus Prestained Protein Ladder (M) (Thermo Scientific, MA, USA). Densitometric analysis performed with Gel Analyzer 19.1 software[2]. The gels were cut to increase the clarity of the presentation. The unedited gels are shown in Supplementary Materials (Figure S14).

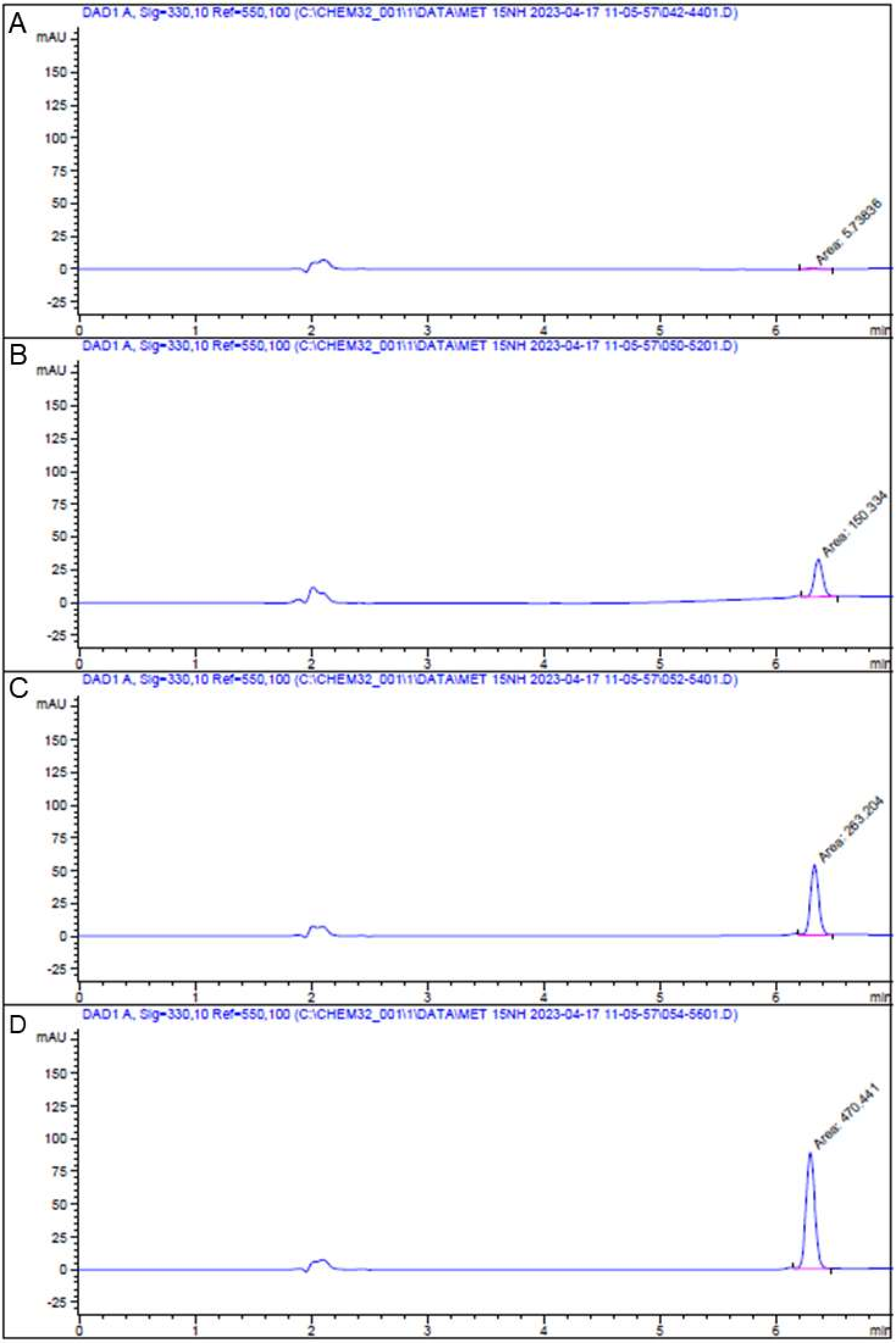

Figure S3. Examples of chromatograms showing peak area of L-HCT-TNB; L-HCT produced in the reaction catalyzed by CaMet15NHp. The reaction mixture consists of 10 nM of enzyme, 0.2 mM PLP, 0.1 mM Na2S, and varying concentrations of OAH: A. 0 mM; B. 20 mM; C. 50 mM; D. 100 mM. Performed via Agilent 1200 Quaternary DAD HPLC System.

Table S1. The change in the surface area under the peak corresponding to HCT-TNB that was observed, depending on the concentration of the OAH substrate. The experiments were performed in triplicates.

L-OAH [mM]	Average	SD
0	5.700	0.308
10	91.575	5.192
20	149.950	4.636
50	269.350	4.675
100	474.775	3.254

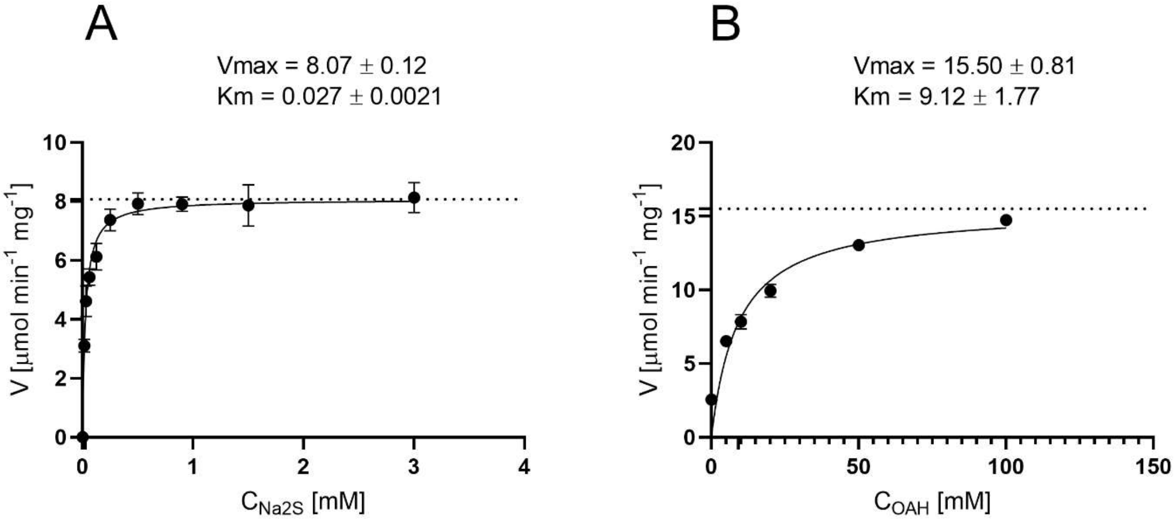

Figure S4. Kinetic parameters of Met15NHp determined for 10 nM enzyme at reaction set for 10 minutes in Tris-HCl pH 8.0 buffer. A K_m and V_{max} were determined for Na_2S at a constant concentration of OAH and PLP equal to 10 mM and 0.2 mM, respectively. B K_m and V_{max} determined for OAH at a constant concentration of Na_2S and PLP equal to 0.1 mM and 0.2 mM, respectively. Calculations were performed with GraphPad Prism 8.0.1 software.

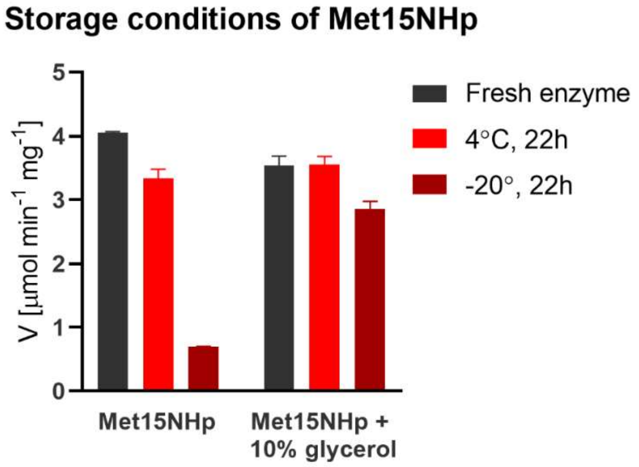

Figure S5. Examination of storage conditions on the activity of Met15NHp. Measurement of the fresh enzyme was performed immediately after purification, after which an enzyme was stored for 22 hours at 4°C or 22°C, with or without supplementation of 20% glycerol.

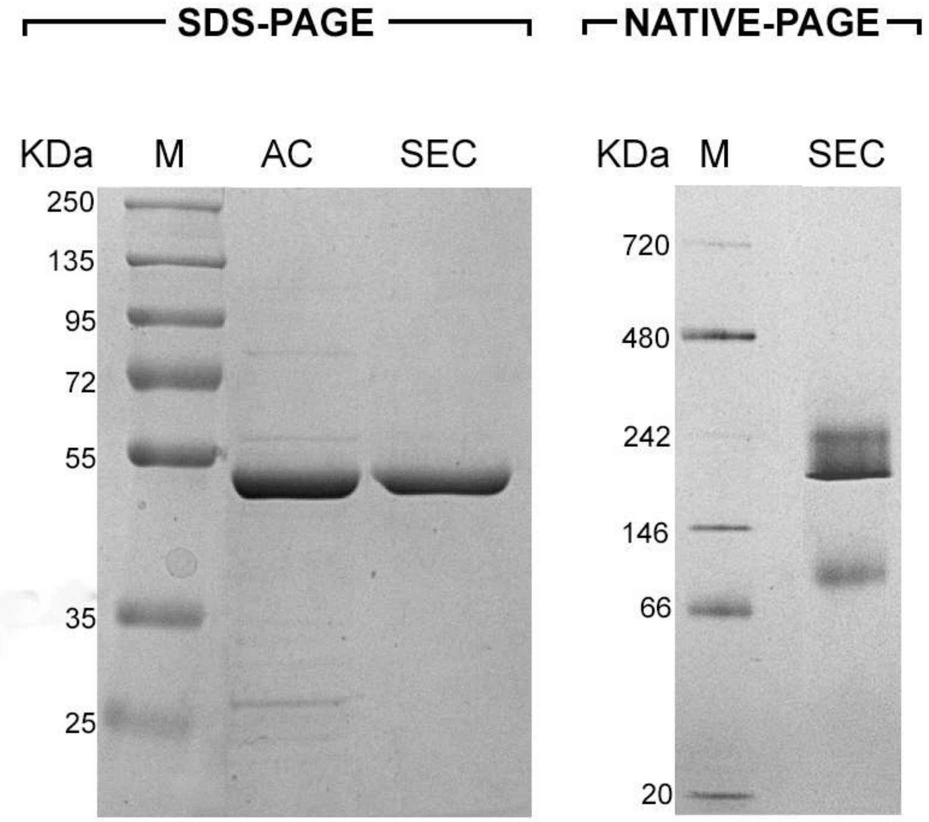

Figure S6. Assessment of Met15NHp molecular mass and oligomeric structure by SDS-PAGE and NATIVE-PAGE electrophoresis. M, marker; AC, affinity chromatography; SEC, size exclusion chromatography. SDS-PAGE electrophoresis was performed at 18 V cm-1 10% gel. Thermo Scientific PageRuler™ Plus Prestained Protein Ladder (M) (Thermo Scientific, MA, USA). NATIVE-PAGE electrophoresis performed at 15 V cm^{-1}; NativePAGE™ Novex Bis-Tris Gel System kit (Invitrogen, MA, USA) 4-16% gel. Thermo Scientific Native MARK™ Unstained Protein Standard (M) (Thermo Scientific, MA, USA). Met15NHp was purified with HisTrap™ Fast Flow 5 mL column and a Superdex 13/300 GL increase column (Cytiva, MA, USA). The gels were cut to increase the clarity of the presentation. The unedited gels are shown in Supplementary Materials (Figure S15).

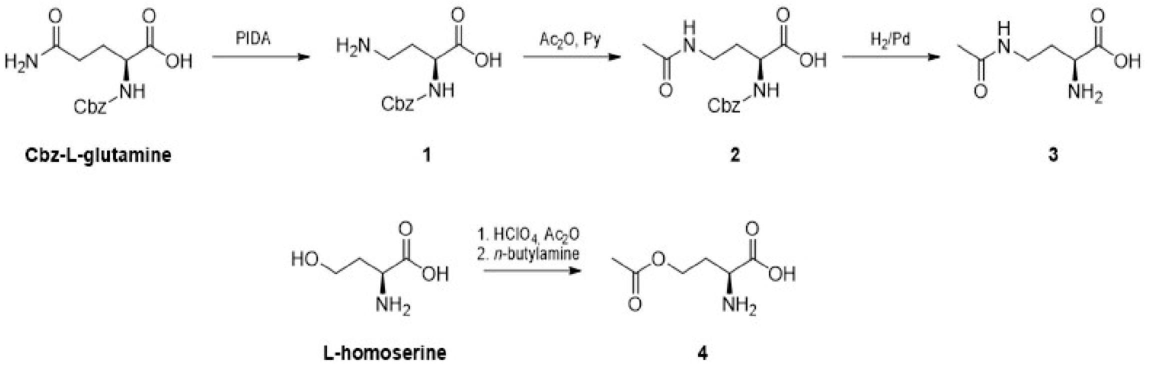

Figure S7. Synthesis of Nᵞ-acetyl-L-2,4-diaminobutanoic acid **3** and *O*-acetyl-L-homoserine **4**. Synthesis of Nᵞ-acetyl-L-2,4-diaminobutanoic acid **3** starts with a reaction between Cbz-L-glutamine and bis(trifluoroacetoxy)iodobenzene (PIDA), resulting in an appropriately protected L-2,4-diaminobutanoic acid **1**[3]. Treatment with an acetic anhydride in the presence of pyridine gives Nᵞ-acetyl product **2**[4]. Final catalytic hydrogenolysis lead to the final compound **3**. *O*-acetyl-L-homoserine **4** is obtained in two subsequent steps[5]. First, L-homoserine is treated with perchloric acid and acetic anhydride in the presence of acetic acid to form intermediate cyclic products. Then, intermediate products are decomposed by the treatment of n-butylamine and the final product is isolated from the reaction mixture[5].

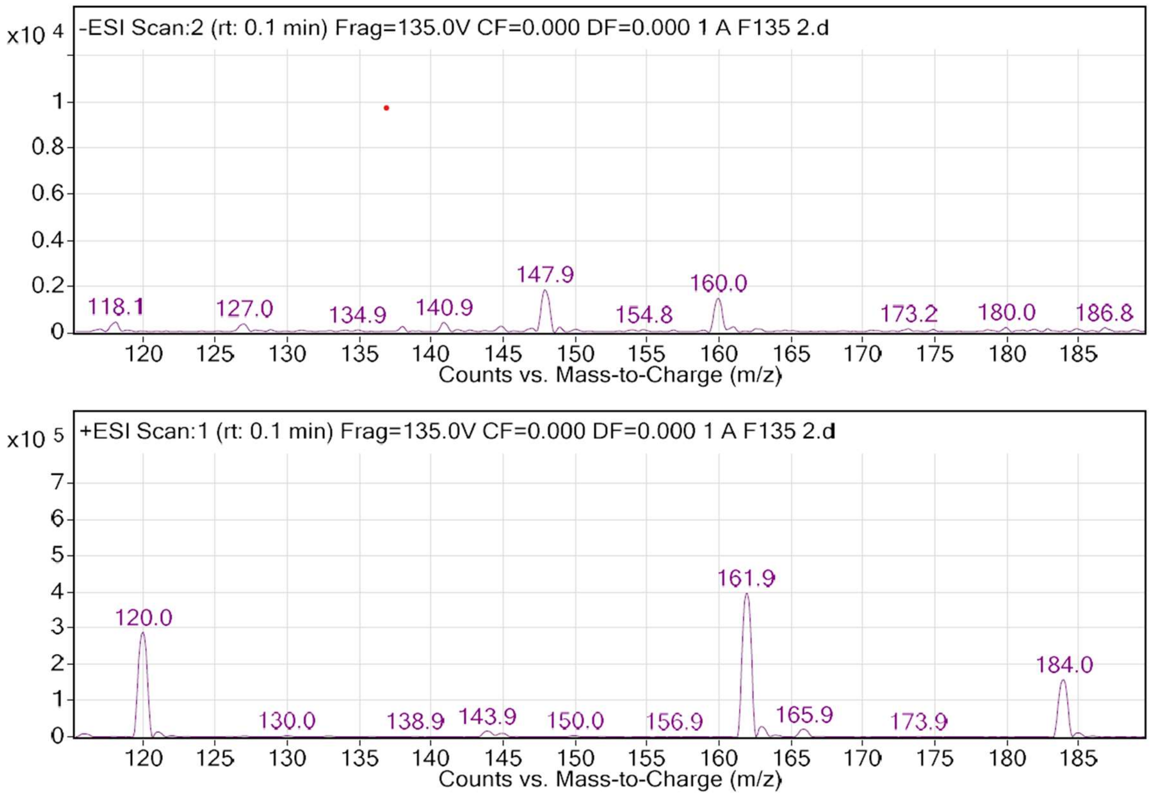

Figure S8. ESI-MS spectrum for *O*-acetyl-L-homoserine.

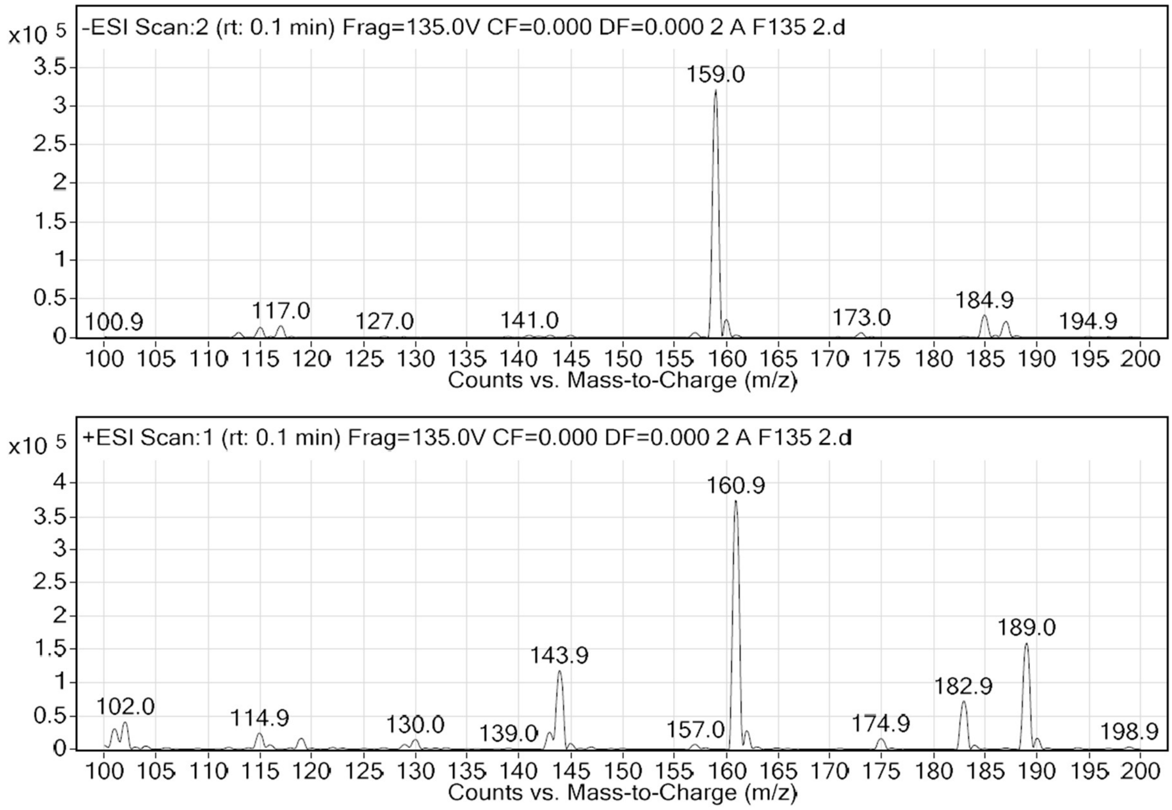

Figure S9. ESI-MS spectrum for N^{γ}-acetyl-L-2,4-diaminobutanoic acid.

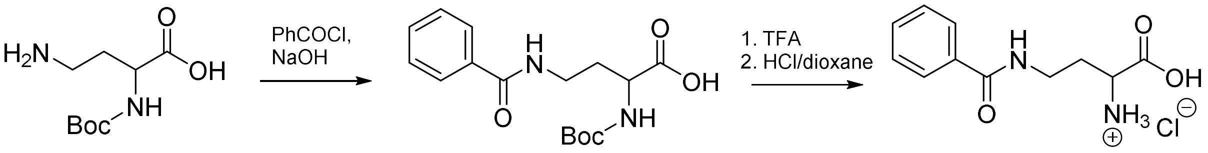

1 **2** **3**

Figure S10. Synthesis of L-4-benzamido-2-aminobutanoic acid chloride (BzDAB) **3.**

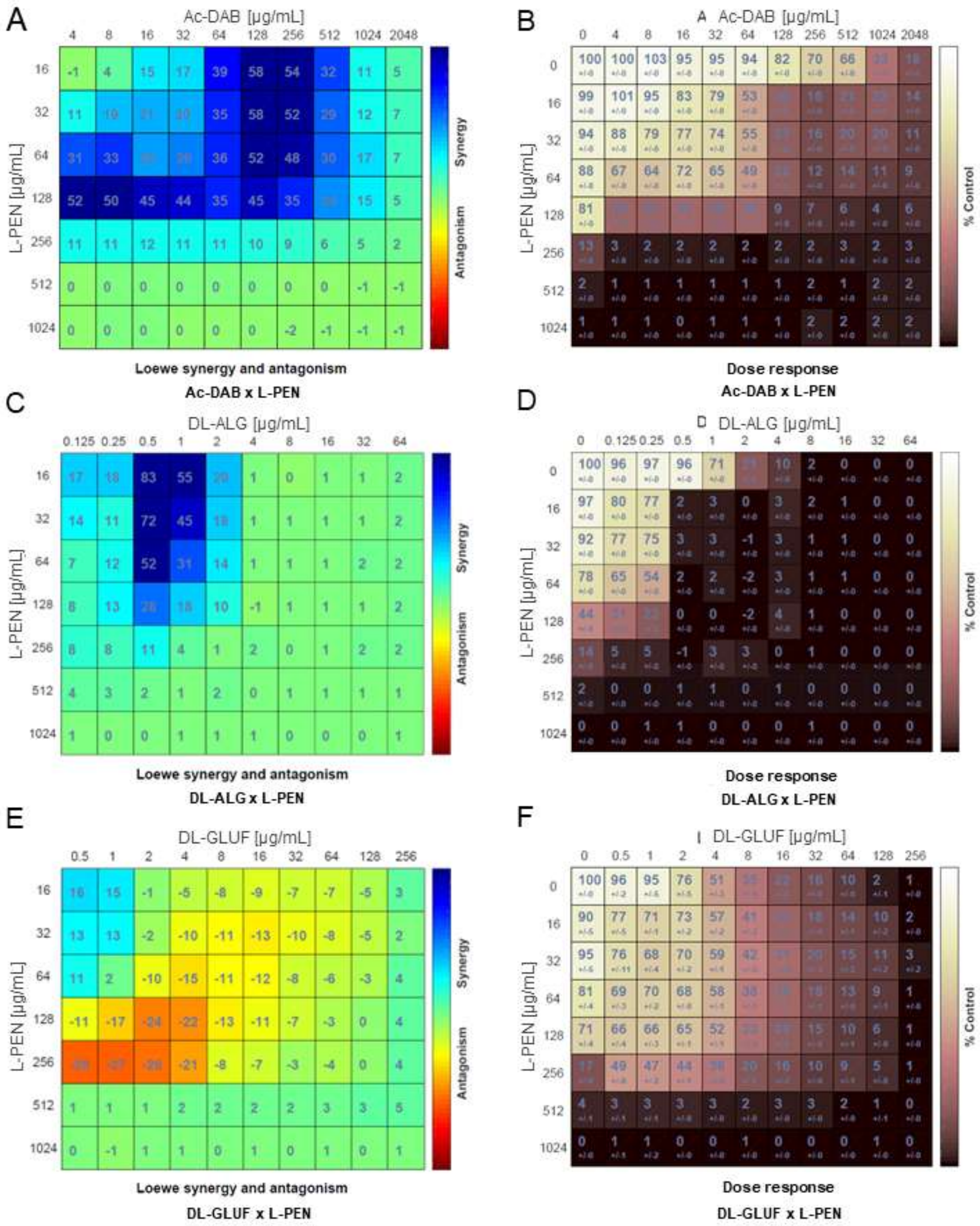

Figure S11. The result of the checkerboard assay of antifungal activity of combinations of L-penicillamine (L-PEN) with L-4-acetamido-2-aminobutanoic acid (AcDAB), DL-2-allyglycine (DL-ALG) or DL-glufosinate (DL-GLUF) in YNB-SG minimal medium against *C. glabrata* ATCC 90030. Combinatory effect scores calculations were obtained according to the Loewe additivity model via Combenefit software26. In Figs A, C and E the results are shown in the form of a heatmap representing the level of synergy at each concentration where synergy (a positive score) is shown in blue color, antagonism (a negative score) is shown in red color and an indifferent effect (score 0) is shown in green color. In Figs. B, D and F, the results are presented as percentages of growth compared to that of the untreated control.

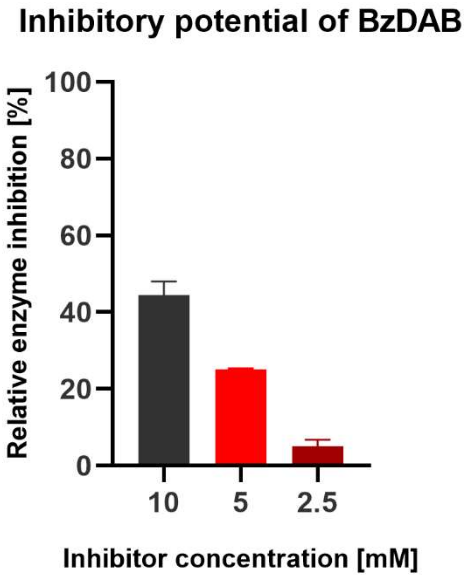

Figure S12. Concentration-dependent inhibitory potential of BzDAB.

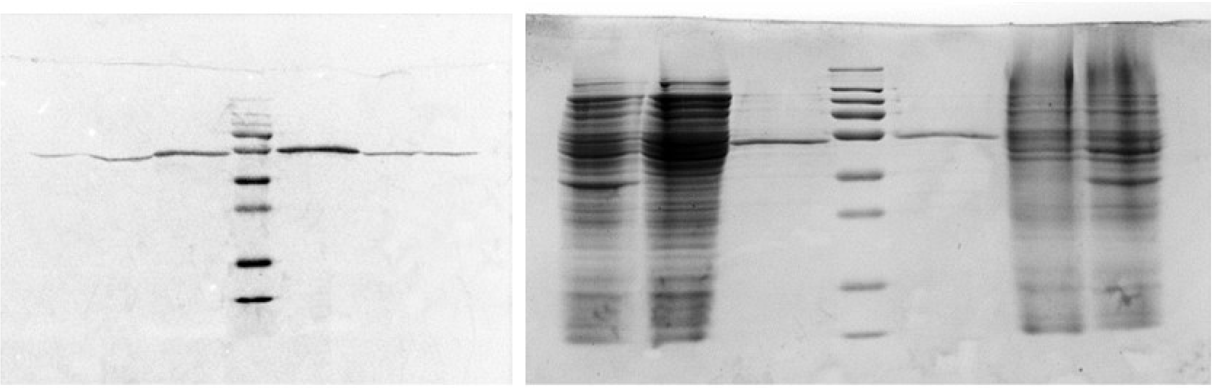

Figure S13. Full length and uncut gels from Figure 3. A. Western Blot analysis of CaMet15NHp and CaMet15CHp purification; B. SDS-PAGE electrophoresis analysis of Met15NHp and Met15CHp purification. Electrophoresis 18 V cm-1 10% gel; H, harvest from protein overproduction; CFE, cell-free extract; PP, purified protein; M, Thermo Scientific PageRuler™ Plus Prestained Protein Ladder. Left A; right B.

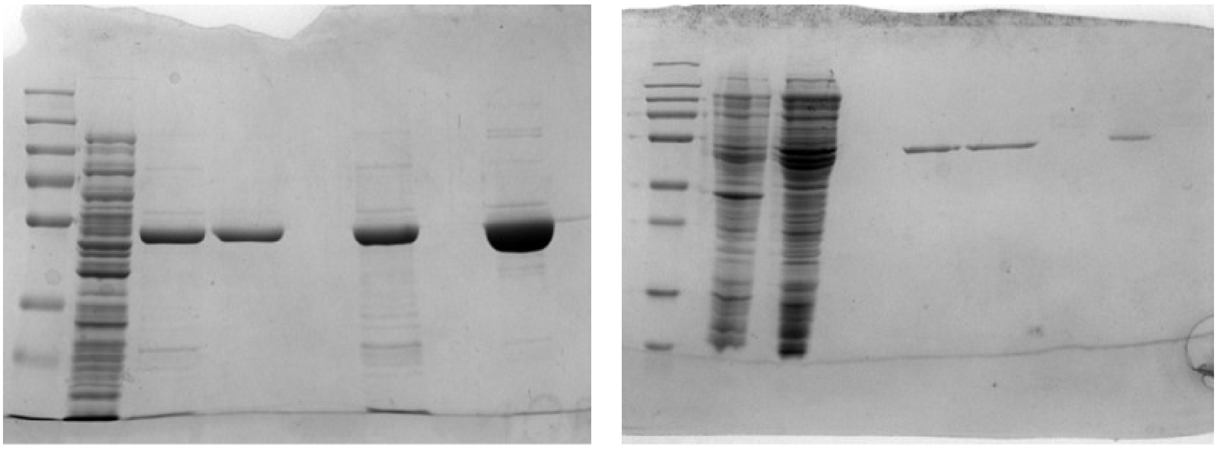

Figure S14. Full length and uncut gels from Figure S2. Left A, B, and C; Right D, E and F.

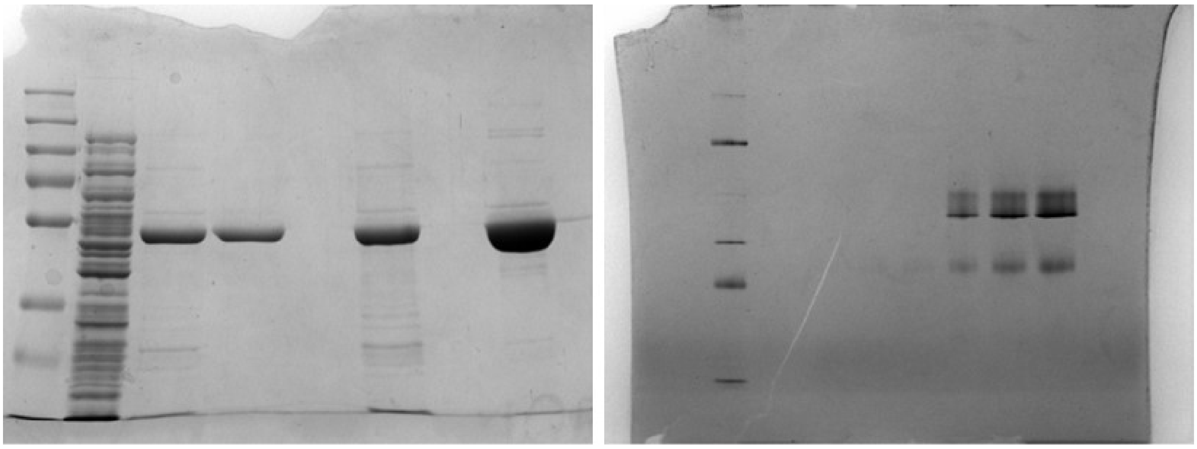

Figure S15. Full length and uncut gels from Figure S6. Left SDS-PAGE; Right NATIVE-PAGE.

References

1. Robert, X. & Gouet, P. Deciphering key features in protein structures with the new ENDscript server. *Nucleic Acids Res.* **42**, W320–W324 (2014).
2. Lazar Jr., I. & Lazar Sr., I. GelAnalyzer 19.1 (www.gelanalyzer.com).
3. Andruszkiewicz, R. & Rozkiewicz, D. An Improved Preparation of N2-tert-Butoxycarbonyl- and N2-Benzyloxycarbonyl-(S)-2,4-diaminobutanoic Acids. *ChemInform* **35**, no-no (2004).
4. Heltweg, B. *et al.* Subtype selective substrates for histone deacetylases. *J. Med. Chem.* **47**, 5235–5243 (2004).
5. Wang, T. *et al.* Efficient synthesis of hydrocarbon-bridged diaminodiacids through nickel-catalyzed reductive cross-coupling. *Tetrahedron Lett.* **58**, 3970–3973 (2017).

Competing interests

The authors declare no competing interests.
